# A Randomised Controlled Trial of YOGa and Strengthening Exercise for Knee OsteoArthritis: Protocol for a Comparative Effectiveness Trial (YOGA Trial)

**DOI:** 10.3390/jfmk7040084

**Published:** 2022-10-12

**Authors:** Ambrish Singh, Dawn Aitken, Steffany Moonaz, Andrew J. Palmer, Leigh Blizzard, Changhai Ding, Stan Drummen, Graeme Jones, Kim Bennell, Benny Antony

**Affiliations:** 1 Menzies Institute for Medical Research, University of Tasmania, Hobart, TAS 7000, Australia; 2 Department of Health Services Research, Southern California University of Health Sciences, Whittier, CA 90604, USA; 3 Research Department, Maryland University of Integrative Health, Laurel, MD 20723, USA; 4 Clinical Research Centre, Zhujiang Hospital, Southern Medical University, Guangzhou 510000, China; 5 Department of Epidemiology and Preventive Medicine, Monash University, Melbourne, VIC 3004, Australia; 6 Department of Physiotherapy, School of Health Sciences, Faculty of Medicine, Dentistry and Health Sciences, University of Melbourne, Melbourne, VIC 3010, Australia

**Keywords:** yoga: strengthening exercise, knee osteoarthritis, comparative effectiveness research, randomised controlled trial

## Abstract

Osteoarthritis (OA) is a common joint disorder for which there is no cure. Current treatments are suboptimal. Exercise is a core treatment for knee OA, with muscle strengthening exercise commonly recommended. Yoga is a mind-body exercise intervention that can improve flexibility, muscle strength, balance, and fitness and potentially reduce symptoms of OA. However, there is a scarcity of robust, high-quality conclusive evidence on the efficacy of yoga in knee OA. We are currently conducting the first randomised comparative effectiveness and cost-effectiveness trial of a yoga program compared with a strengthening exercise program in patients with symptomatic knee OA. This study protocol describes the design and conduct of this trial. The YOGA study is a phase III, single-centre, parallel, superiority, randomised, active-controlled trial which will be conducted in Hobart, Australia. One hundred and twenty-six participants (63 in each arm) aged over 40 years with symptomatic knee OA will be recruited from the community and randomly allocated to receive either a 24-week yoga program (3×/week) or a strengthening exercise program (3×/week). The primary outcome will be change in knee pain over 12 weeks, assessed using a 100 mm visual analogue scale (VAS). The secondary outcomes include change in knee pain, patient global assessment, physical function, quality of life, gait speed, biomarkers, and others over 12 and 24 weeks. We will also assess whether the presence of neuropathic pain moderates the effects of yoga compared to strengthening exercise. Additional data, such as cost and resource utilization, will be collected for the cost-effectiveness analysis. The primary analysis will be conducted using an intention-to-treat approach. Adverse events will be monitored throughout the study. Once completed, this trial will contribute to the knowledge of whether yoga can be used as a simple, effective, low-cost option for the management of knee OA, thus saving economic costs in the healthcare system.

## 1. Introduction

Osteoarthritis (OA) is the most common joint disorder in the world and affects over 2.2 million people in Australia, with a direct health expenditure of Australian dollar (AUD) 3.5 billion in 2015–2016, representing 28% of overall expenditure on musculoskeletal conditions and 3% of total disease expenditure [1]. Despite its substantial disease burden, no approved disease-modifying treatments are available for OA [2]. Traditional pharmaceutical therapies such as analgesics, corticosteroids, and non-steroidal anti-inflammatory drugs (NSAIDs) are, at best, only modestly effective for pain and show no or deleterious effect on joint structures, with end-stage OA treated with costly joint replacements [3]. There is evidence that non-pharmacological treatment, such as exercise (aerobic, muscle-strengthening), reduces pain and improves function and quality of life (QoL) in OA populations [4,5,6].

Yoga is a mind–body exercise constituting physical postures (asanas), breathing practices (pranayama), meditative mental focus (dhyana), and relaxation as the four standard components [7]. With the increasing popularity of yoga globally, it is becoming a more common exercise to achieve and maintain well-being and health [8,9,10]. Yoga has been used clinically as a therapeutic intervention for improving pain, stiffness, swelling, and mobility in older adults and is recommended by some clinical guidelines for individuals with knee OA [11,12,13,14,15,16]. However, the current guidelines for the treatment of knee OA suggest a “conditional recommendation” for yoga as an adjunctive form of exercise and only for short-term management. The guidelines highlight the lack of evidence and the poor quality of the existing evidence [17]. Similarly, a recent systematic review found that yoga may be effective for improving pain, function, and stiffness in knee OA; however, due to the low methodological quality and potential risk of bias, only a weak recommendation can be made for the use of yoga in knee OA [18]. A network meta-analysis assessing the effects of various non-pharmacological interventions on pain relief in patients with OA reported yoga as the most effective intervention for patients and with a high adherence rate [19]. Another recent systematic review synthesising evidence of effectiveness and safety of yoga for OA patients identified nine randomised controlled trials (RCTs) of low methodological quality and potential risk of bias and provided a weak recommendation to support the use of yoga in knee OA [18]. Most of these studies compared yoga with usual care, and only a few trials compared yoga with another active intervention [18]. On the other hand, among nonpharmacological treatments of OA, strengthening exercise is strongly recommended for the treatment of knee and hip OA by all the major guidelines [3]. Hence, given the relative scarcity of robust, high-quality conclusive evidence for yoga in OA, we aim to evaluate the effect of yoga compared to strengthening exercise in patients with knee OA.

The mechanisms of action of yoga in OA may extend beyond the exercise component. The physical component of yoga provides exercise that is consistent with recommendations for knee OA, while the mind component of yoga has the potential to increase psychological well-being and reduce stress, improve function, and alleviate OA-associated pain [20]. A previous trial has reported that sitting meditation without any physical activity component improved function in OA patients [21]. We have shown that OA is a heterogeneous disease with multiple phenotypes that may require somewhat different approaches for each patient in order to optimise treatment [22,23,24,25]. Pain phenotypes can be defined as subtypes of OA that share a distinct underlying pain mechanism. The pain phenotypes of OA are complex and may be influenced by psychological factors (e.g., depression, pain-related fear, and anxiety) [26,27,28] and neural sensitisation [29,30]. The discrepancies observed between symptom severity and structural abnormalities of knee OA and the existence of persistent pain even after total knee replacement also indicates these factors [31,32]. There is increasing clinical evidence to suggest that neuropathic pain may contribute to the pain experience in OA patients [33]. Given that central sensitisation plays a role in chronic neuropathic pain, centrally acting physical exercises may be effective [25,29,34]. Preliminary studies in OA patients with a neuropathic pain phenotype have demonstrated that treatment with non-standard analgesics, such as tricyclic antidepressants and gabapentinoids, may be more effective than conventional treatments [25]. Therefore, we also aim to conduct a priori subgroup analyses to assess whether the effects of yoga are greater in the subgroup of patients with neuropathic pain, as assessed by the painDETECT questionnaire, than in those without neuropathic pain.

This paper presents the detailed protocol of this first comparative effectiveness and health economic analysis of a randomised controlled trial of yoga compared to strengthening exercise in adults with knee OA. The study results will be published at the completion following the Consolidation of Standards for Reporting Trials guideline [35]. This study will fill important evidence gaps and generate clinical insights to inform decision making for the management of knee OA.

## 2. Study Aims and Hypotheses

The primary aim of this 24-week RCT is to compare the effectiveness of a 12-week group-based yoga program (2×/week of in-person group-based yoga plus 1×/week of home-based yoga) on reducing knee pain (assessed using VAS) over 12 weeks in knee OA patients compared to a 12-week group-based strengthening exercise program (2×/week of in-person group-based exercise plus 1×/week of home-based exercise). We hypothesise that yoga will outperform strengthening exercise, and we aim to demonstrate that, compared with strengthening exercise, yoga may be an effective and cost-effective therapy for managing pain and reducing the functional limitations that impact the QoL in patients with knee OA. A secondary aim is to assess whether the presence of neuropathic pain moderates the effects of yoga compared to strengthening exercise.

## 3. Method and Analysis

### 3.1. Trial Design

**Figure 1:**
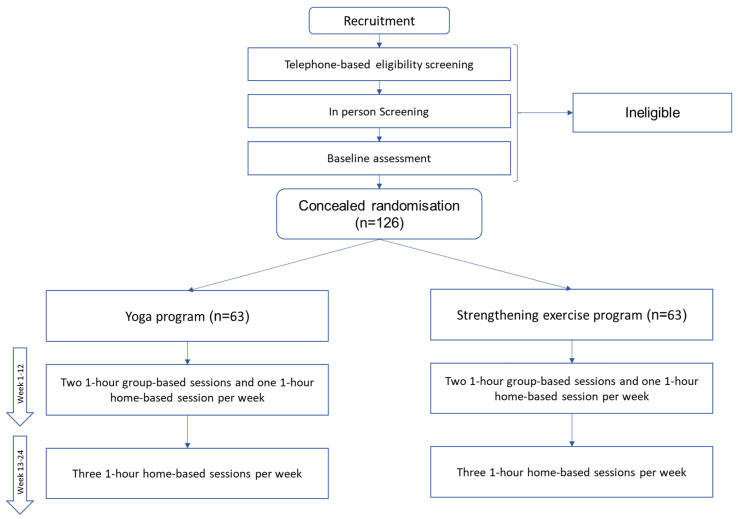
Trial design.

The YOGA trial is designed as a parallel, randomised, single-centre, single-blinded, active-controlled, two-arm clinical trial with a 1:1 allocation ratio. The protocol is described using the Standard Protocol Items: Recommendations for Interventional Trials (SPIRIT) guidelines, and the study results will be reported using the CONSORT statement (Figure 1).

### 3.2. Study Participants

We will recruit 126 knee OA participants with knee pain from the southern Tasmania region of Australia. Community-based recruitment will be accomplished through collaboration with general practitioners, specialist rheumatologists, orthopaedic surgeons, physiotherapists, and advertising through radio, TV, newspaper, social media, community notice boards, hospital notice boards, community newsletters, local press, physiotherapy practices, and exercise physiologists in the region. Previous clinical trial participants who expressed interest in future studies will be contacted.

Inclusion criteria:

1. Aged ≥40 years, both males and females;
2. Knee pain on most days for at least six months;
3. Average VAS knee pain intensity of ≥40 mm in the last month;
4. Meet the American College of Rheumatology (ACR) clinical criteria for the diagnosis of knee OA;
5. Be willing to participate in a group yoga program or group strengthening exercise program two times per week for the first 12 weeks and can attend on the days/times of the week that scheduled classes are running.

Exclusion criteria:

1. Patients currently or in the past three months engaged in strengthening exercise or yoga programs for the treatment of any disease;
2. Other forms of inflammatory arthritis (especially rheumatoid arthritis and gout);
3. A significant knee injury that required treatment within the last six months;
4. Arthroscopy or open surgery in the index knee in the last six months or planned in the next 6–8 months;
5. Partial or total knee replacement;
6. Injections of corticosteroids (last three months) or hyaluronic acid (last six months) in the index knee;
7. Pregnancy or breastfeeding;
8. Currently participating in any other drug/device/exercise clinical trial related to OA;
9. Presence of any serious medical illness or condition that may preclude a 24-week follow up;
10. Any condition that precludes safe participation in exercise (i.e., fails the safety for exercise clearance; see below for the procedure for this);
11. Unable to walk without a gait aid;
12. Inability to provide informed consent in English;
13. Plan to start an exercise-based treatment program (e.g., GLA:D) or another new treatment for knee OA in the next six months;
14. Planned absences (e.g., trips away) of >2 weeks maximum during the 12-week period.

### 3.3. Screening

Volunteers will be screened over the telephone for general eligibility and invited to the trial centre for a screening visit which comprises symptom assessments, medication history questionnaires, safety for exercise clearance, and clinical assessment for American College of Rheumatology (ACR) criteria. In the case of bilateral eligible knees, the most symptomatic knee will be the study knee. In the case of bilaterally eligible knees with equal symptoms, the dominant knee will be studied. The dominant knee is the knee with which the individual steps first when initiating gait.

### 3.4. Safety for Exercise Clearance

During the screening, the Adult Pre-exercise Screening Tool (stage 1) will be administered. The tool was developed by Exercise and Sports Science Australia (ESSA), Fitness Australia, and Sports Medicine Australia (SMA) to identify those individuals with a known disease or signs or symptoms of a disease who may be at a higher risk of an adverse event during physical activity/exercise. This approach of screening patients for exercise participation aligns with the recommendation from the American College of Sports Medicine. Participants who mark “yes” to any of these questions will need to provide a letter from their GP stating that they approve of their participation in the yoga trial.

### 3.5. Randomisation and Blinding:

Allocation of participants in a 1:1 ratio to either the yoga program or strengthening exercise program will be based on computer-generated random numbers prepared by a statistician with no involvement in the trial. The administering institute will maintain the code-break for the full randomisation schedule. Allocation concealment and blinding will be ensured by having two research nurses coordinate the trial. Clinical assessments will be taken by the research nurse blinded to the group allocation. Due to the nature of the intervention, participants will not be blinded to the treatment allocation. Thus, patient-reported outcomes will not be blinded.

### 3.6. Treatments

Overall, 126 eligible participants will be randomly allocated to a 24-week group-based yoga program or strengthening exercise program (each with two 1 h in-person group-based sessions and one 1 h home-based session per week for the first 12 weeks + 3 home-based sessions per week for another 12 weeks). Group sessions will be led by physiotherapists/exercise physiologists/yoga teachers for the first 12 weeks. Trainers will lead a group of approximately 10 participants. Participants will be instructed to continue their yoga or strengthening exercise at home for another 12 weeks. Using REDCap, weekly emails will be sent to participants to promote adherence to the home-based program, and video instructions will be given to each participant to support the home-based program.

An evidence-based yoga program designed by a yoga expert and researcher—Dr. Steffany Moonaz from the Southern California University of Health Sciences, USA—will be delivered by yoga teachers (RYT-200 or greater) [36]. The yoga teachers will undergo orientation to the intervention and will be trained in advance with questions/concerns addressed and periodic support during the intervention period as needed. Each yoga class will begin with a breathing exercise (*pranayama*) and chanting (10 min), warm-up, and moving sequences (*asanas*, 40 min). Classes will end with deep relaxation (*savasana*) and meditation (10 min). Classes will include an application of yoga philosophy (*yama* and *niyamas*) and replacing negative thoughts with positive ones (*pratipaksa bhavanam*) [36]. The practices will be modified for the needs and limitations of each participant. Participants will also be given access to online yoga practices developed for this population. Details about this intervention is in Supplementary Materials S1.

An evidence-based lower-limb strengthening exercise therapy regimen designed by an expert and researcher—Professor Kim Bennell from the University of Melbourne, Australia—will be delivered as a group-based progressive strengthening exercise program by physiotherapists/exercise physiologists. Each class will begin with an assessment (5 min) followed by warm-up (5 min) and lower-limb strengthening exercises (run as a circuit class for 45 min), and in the end, five minutes of cool down. In addition, participants will be allowed to study their non-study leg by alternating both legs, with the instruction to start with the study leg. If they did not have enough time to finish, at least the study leg would have had 3 sets of 10. Detail about this intervention is in Supplementary Materials S2.

### 3.7. Safety

We will monitor the adverse events (AEs) throughout the study. Yoga or exercise teachers and the unblinded research assistant will monitor AEs. AEs will be defined as any problem that lasts for >2 days and/or causes the participant to seek other treatment. The commonly possible AEs from this study include temporary muscle stiffness, elevated knee pain or pain at other sites, and falls or other exercise-related injuries. There is a very rare chance of an exercise-induced heart attack or sudden death. AEs will be noted throughout the study, and the chief investigator (CI) will be notified of any serious events within 24 h.

### 3.8. Primary Outcome

Change in overall average knee pain assessed by visual analogue scale (VAS) over 12 weeks.

Pain will be evaluated at each time point using a 100 mm VAS and asked: “On this line, how would you rate your overall average knee pain in the last one week?” For knee pain assessed using VAS, terminal descriptors will be 0 mm = no pain to100 mm = worst pain possible.

### 3.9. Secondary Outcomes: The Overall Change from Baseline to Week 12 and Overall Change from Baseline to Week 24 Are Separate Outcomes

1. Change in VAS knee pain over 12 weeks in patients with painDETECT > 12;
2. Change in VAS knee pain over 24 weeks;
3. Change in Western Ontario and McMaster Universities Osteoarthritis Index (WOMAC) knee pain, WOMAC stiffness, and WOMAC knee function assessed using VAS over 12 weeks and over 24 weeks;
4. Change in core physical function as assessed by 30 s chair stand test, 40 m fast walk test, and stair climb test over 12 and over 24 weeks.

Core physical function measures: Objective measures, including a 30-second chair stand test, 40 m (10 m × 4) fast-paced walk test, and stair climb test, will be performed as recommended by the Osteoarthritis Research Society International (OARSI) guidelines for clinical trials at baseline and week 12 using the same equipment and at the same location. The total number of chair stands in 30 s, time to complete 40 m walk with three turns, and time to ascend and descend a nine-step stair (20 cm step height and handrail) will be recorded. [37,38]

1. Change in biomarkers (urinary CTX-II, serum COMP, and serum hyaluronan) and systemic inflammatory markers (hs-CRP, IL-6, TNF-a) over 12 and over 24 weeks;
2. Change in Patient Health Questionnaire (PHQ-9) scales over 12 and over 24 weeks.

Patient Health Questionnaire (PHQ-9)*:* Depression may be an effect modifier. It will be assessed using the Patient Health Questionnaire (PHQ-9) at weeks 0, 12, and 24. The PHQ-9 is a validated tool for screening, diagnosing, monitoring, and measuring the severity of depression. It consists of nine questions based on the Diagnostic and Statistical Manual of mental disorders (DSM-IV) criteria for the diagnosis of major depressive disorders in patients with medical illnesses [39].

1. Change in patient global satisfaction score (assessed using a 100 mm VAS) over 12 and over 24 weeks [40]. Patients will be asked, “Considering all the ways in which illness and health conditions may affect you at this time, please indicate on the line below how you are doing ?”, along with a 0–100 VAS, where 0 is very well, and 100 is very poor;
2. Change in neuropathic pain, as assessed by the painDETECT questionnaire, over 12 and over 24 weeks.

PainDETECT questionnaire: It uses a combination of VAS, Likert-type questions, and body diagram to ask about everyday frequency of symptoms such as “electric shocks” or “painful light touch” [41].

1. Change in leg muscle strength will be assessed by leg muscle strength dynamometry at the lower limb (involving both legs simultaneously) over 12 and over 24 weeks.

Leg muscle strength*:* The muscles measured in this technique are mainly the quadriceps and hip flexors. Participants will stand on the back of a dynamometer platform with their backs against a wall and knee flexed to 115°. The previously published repeatability estimate (Cronbach’s α) for this method is 0.91 [42].

2. Change in gait characteristics such as gait speed, step length, double support time, step width, and step time from baseline to week 12 and baseline to 24 weeks.

Gait characteristics*:* Gait characteristics will be assessed using the footfalls recorded on the GAITRite system, a 4.6 m computerised walkway. Measures of gait characteristics such as gait speed (cm/s), step length (cm), double support time (ms), step width (cm), and step time (ms) will be directly obtained from the GAITRite software;

3. Change in physical activity will be assessed, and the participants will wear accelerometers for a week before the start of the intervention at 12 weeks and 24 weeks.

Physical activity*:* We will assess the physical activity of participants by using waist-worn ActiGraph^®^ wGTX3-BT (Firmware 1.9.2) activity monitors (ActiGraph LLC, Fort Walton Beach, FL, USA) if participants had at least 4 days of 10 h wear-time, following settings as recommended by Migueles et al. [43];

4. Self-reported adherence to the yoga or strengthening exercise program from baseline to 12 and baseline to 24 weeks will be assessed using an online logbook and defined as the percentage of prescribed sessions undertaken;
5. Change in body fat will be assessed using bioelectrical impedance analysis (BIA) (BIA analyser, Quantum II, RJL Systems, MI, USA) at baseline, week 12, and week 24. We will assess fat-free mass, percentage of fat-free mass, fat mass, and percentage of fat mass;
6. The OARSI-OMERACT responder criteria: This will be employed to generate a responder categorical variable (0 = non-responder, 1 = responder) based on improvement in WOMAC pain, function, and patient’s global assessment;
7. Pain medication use: There will be no constraints with regard to the use of analgesic medications. All participants will be allowed to continue taking the medications they are taking at their screening visit for the duration of the trial. Participants will be asked to keep medications as stable as possible, but if a participant requires an increase in analgesics, this will be permitted, and the reason for the dose increase and the dose used will be documented. Any medication changes will be documented with the reason, drug name, and dose. Medication change will be classified as commenced or increased, discontinued, or decreased, or stable use or non-use, and the change in total number of pain medications. A rescue medication, paracetamol, will be provided if the participant requests it. Medication use will be recorded at baseline and during each follow-up period.

## 4. Health Economics Outcomes (Secondary Outcomes):

Health-related quality of life (HRQoL) and health state utility (HSU) will be assessed using the Assessment of Quality of Life (AQoL-8D) and the EuroQol 5 dimensions (EQ-5D-5L) instruments. In combination with the clinical data, these data will be used to conduct a cost-effectiveness analysis of the yoga program versus the strengthening exercise program [44,45]. We will test the hypothesis that the yoga program will be more cost-effective than the strengthening exercise program.

The health economic outcomes to be collected as the secondary outcome will include:

8. Change in concomitant medications (assessed using self-reported medication history questionnaire) from baseline to 12 and baseline to 24 weeks and associated costs;
9. Change in QoL (assessed by AQoL-8D and EQ-5D-5L) from baseline to week 12 and baseline to 24 weeks [46,47,48];
10. Change in health resource utilisation (assessed using a self-reported questionnaire) at 12 weeks.

Additional details on these measures are provided in Table 1 and the section describing cost-effectiveness analysis.

### 4.1. Data Integrity and Management

We will collect all data using a custom-built and secure REDCap database hosted by the University of Tasmania. We will keep stored the paper copies of participant questionnaires in locked filing cabinets with restricted access. We will keep the electronic data on password-protected servers, separating the identifying and non-identifying information. We will keep the codes, with linking data to identify participant information, separately from the study data, under password security and with controlled access. The access to identifiable information will only be available to the members of the study team who need to contact trial participants, enter data, or perform data-quality control. Daily backups of all electronic data will be done to reduce the risk of lost data.

### 4.2. Sample Size Calculation

Our power calculations are based on a sample size that can document the superiority of yoga compared to strengthening exercise by an amount that meets the minimal clinically important difference (MCID). Based on the MCID of 15 mm in VAS pain (a numerical scale ranging from 0 (no problem) to 100 (maximum problem)), the two-sided significance level of 0.05, 90% power, and SD of VAS pain change 22.5, we will need a total of 98 participants. Assuming a scenario of around 20% dropout, we will need 126 participants.

The above sample size will allow us to detect a non-inferiority of yoga with strengthening exercise. Non-inferiority would be declared if the mean change in knee pain (assessed using VAS) in the yoga group was not significantly worse than the mean change in the strengthening exercise group, within a pre-stated margin of non-inferiority (∆), in this case, set at 10 mm. Overall, 126 participants will give us 80% power to detect the non-inferiority of yoga compared with strengthening exercise.

### 4.3. Statistical Analyses

The comparisons of pain and other continuous outcomes will be done employing a repeated-measures mixed model incorporating terms of treatment, time, and respective baseline values as covariates. The treatment effect will be calculated by the intervention by time interaction followed by the main effects model with only group and time. We will also assess the effects of potential confounders or interaction with treatment by covariates, including age, sex, BMI, disease severity, comorbidities, health status, and use of pain medications. A two-sided *p*-value less than 0.05 will be considered to indicate the statistical significance, and the results will be shown as the between-group differences with 95% confidence intervals (95% CIs) of the differences.

We will explore the role of clustering effects in the trial by performing a sensitivity analysis examining the implications of clustering by the instructor by adding an instructor random effect to our mixed-effects model.

We will also perform an exploratory analysis to investigate whether the presence or absence of neuropathic pain, as assessed by the painDETECT questionnaire, is a potential moderator that influences response to treatment for the primary outcome at 12 weeks. To assess the moderation of the effect by painDETECT (binary moderator), an interaction term between the randomised group and the potential moderator will be included in outcome regression models.

### 4.4. Cost-Effectiveness Analysis

We will perform the cost-effectiveness analysis (CEA) by measuring the costs and benefits in the yoga program group and the strengthening exercise program group. Mean differences in total costs and benefits between the yoga program and strengthening exercise program at 12 weeks will be calculated. Incremental cost-effectiveness ratios (ICERs) will be determined by dividing the difference in total costs by the difference in total benefits (assessed as QALY) for both groups (equation below). We will also conduct a subgroup analysis based on the degree of knee pain at baseline, adherence to the yoga program, sex, and socioeconomic status.

#### 4.4.1. Measurement of Costs

The health questionnaires completed by participants at baseline and 12 weeks will provide data on “health service use”. These data will include visits to GPs, practice nurses, and any other health professionals (e.g., physiotherapists) for the treatment and/or management of knee OA. To estimate total costs, we will assign unit costs for each visit to a health care professional. Unit costs will be obtained from national published sources such as the appropriate Australian Annual Medicare Statistics providing health service unit cost and the Indexation of Medicare Benefits Schedule [49].

#### 4.4.2. Measurement of Benefit

As described under secondary outcomes, AQoL-8D and EQ-5D-5L will be used to determine HSU for each participant. Mean scores and measures of dispersion will be calculated for both groups. Quality-adjusted life-years (QALYs) will be calculated using two approaches: change from baseline (CfB) and area under the curve (AUC) approach with/without linear regression [44,45].

#### 4.4.3. Uncertainty and Sensitivity Analysis

A summary measure of the uncertainty of costs and effects will be presented using cost-effectiveness acceptability curves (CEAC). The CEAC will show a range of probabilities of an intervention being cost-effective at different ceiling thresholds (i.e., a maximum amount that decision-makers are willing to pay for a unit of benefit). To confirm the robustness of our results, a series of sensitivity analyses will be conducted to explore the variability in estimating cost-effectiveness.

$$ICER=\frac{mean\;cost\;yoga\;program\;group-mean\;cost\;physical\;therapy\;group}{mean\;QALY\;yoga\;program\;group-mean\;QALY\;physical\;therapy\;group}$$

## 5. Discussion

Osteoarthritis is one of the major causes of pain and disability globally, and knee OA is the most common form of lower limb OA. Currently, there is no disease-modifying treatment option available for the management of OA. Consequently, OA imparts a substantial and increasing health burden, with notable implications for individuals and the healthcare systems globally [50].

Current pharmacological treatments for patients with symptomatic OA are palliative and primarily focus on pain relief. The non-pharmacological treatments such as exercise therapy (aerobic, muscle-strengthening, etc.) improve pain, function, depression, and QoL in people with OA [51]. While it is established that exercise is a core treatment for OA, the evidence for some forms of popular exercise such as yoga is sparse. Increasing evidence demonstrates that neuropathic pain may contribute to the pain experience by OA patients. Yoga is a mind–body therapy, and the physical component of yoga provides exercise that is consistent with recommendations for knee OA [14,52], while the mind component has the potential to increase psychological well-being and reduce stress [53,54], which can influence central sensitisation. Therefore, it is possible that OA patients may show greater benefit from centrally acting non-pharmacological therapies such as yoga [29,34,55,56,57] than from other common forms of exercise such as muscle strengthening and that this effect could be greater in those with neuropathic pain.

Only a few studies have evaluated the effect of yoga in patients with OA. Current guidelines for the treatment of knee OA conditionally recommend yoga as an adjunctive form of exercise and only for short-term management [11,14,17]. The guidelines highlighted the lack of evidence and the poor quality of the existing evidence. A systematic review of yoga intervention studies (including RCT and non-RCT) in OA reported a significant effect on pain and mobility. However, they reported a lack of data on QoL and mental health [58]. Small sample sizes, shorter duration of follow-up, lack of an active comparator, and poor methodological quality of previous studies prevent definitive conclusions about the use of yoga in knee OA.

Given the relative scarcity of robust, high-quality conclusive evidence for yoga in knee OA, we are conducting randomised comparative effectiveness and cost-effectiveness trial to generate high-quality evidence about the comparative effectiveness, safety, and cost-effectiveness of yoga compared to commonly prescribed strengthening exercise in patients with symptomatic knee OA. [59] When completed, the trial will be the first study to compare yoga to a strengthening exercise program for the management of symptomatic knee OA. We will also explore whether the presence of neuropathic pain modifies the outcome of yoga compared to strengthening exercise.

Thus, successful completion of the proposed trial will contribute to the knowledge of whether yoga can be used as a simple, effective, low-cost option for the management of knee OA, thus saving economic costs in the healthcare system. The study will be completed by December 2022.

## Figures and Tables

**Table 1:** SPIRIT (Standard Protocol Items: Recommendations for Interventional Trials) diagram of enrolment, interventions, and assessments for the YOGA trial.

Items/Variables	Screening	Baseline (week 0)	Week 4	Week 8	Week 12	Week 16	Week 20	Week 24
Informed consent	x							
Randomisation		x						
Safety for exercise clearance	x							
ACR clinical criteria for knee OA	x							
Medicare number		x						
Clinical measures								
Blood (stored for cartilage/synovium/ inflammatory markers)		x			x			x
Core physical function tests		x			x			x
Leg muscle strength test		x			x			x
Height and weight		x			x			x
Gait characteristics		x			x			x
Body composition (using BIA)		x			x			x
Physical activity (using accelerometers)		x						x
Questionnaires								
Knee pain VAS	x	x	x	x	x	x	x	x
Knee WOMAC		x	x	x	x	x	x	x
PainDETECT		x			x			x
PHQ-9		x			x			x
Patient global evaluation		x	x	x	x	x	x	x
Pain medication use/change in use		x	x	x	x	x	x	x
Health Economics Outcomes: Medication cost diary Health service utilisation (visit to GP, practice nurses, and any other health professionals (e.g., physiotherapists)) Employment/days off work Concession/health care card Private health insurance Transport and specialised equipment costs		x			x			
Safety (AEs)			x	x	x	x	x	x
EQ-5D and AQoL-8D		x			x			x
Consent to contact for future studies								x
Early withdrawal information		As required						

ACR, American College of Rheumatology; AE, adverse events; AQoL-8D, Assessment of Quality of Life-8 dimension; BIA, Bio-Electrical Impedance Analysis; EQ-5D, European Quality of Life Five Dimension; GP, general practitioner; OA; osteoarthritis; PHQ-9, Patient Health Questionnaire-9; VAS, visual analogue scale; WOMAC, Western Ontario and McMaster Universities Osteoarthritis Index.

## Data Availability

Not applicable for this type of manuscript. The study database is yet to be analysed, and further information will be accessible from the corresponding author after the completion of the trial.

## Supplementary Materials

The following supporting information can be downloaded at: https://www.mdpi.com/article/10.3390/jfmk7040084/s1, Supplementary Material S1: description of Yoga program, and Supplementary Material S2: description of Strengthening Exercise.
