# Supplementary material for: A Randomised Controlled Trial of YOGa and Strengthening Exercise for Knee OsteoArthritis: Protocol for a Comparative Effectiveness Trial (YOGA Trial)
Source: J Funct Morphol Kinesiol. 2022 Oct 12;7(4):84. doi: 10.3390/jfmk7040084 (PMC9624302; doi:10.3390/jfmk7040084)
Supplement: Supplementary file 1 [file jfmk-07-00084-s001.zip › Supplementary Materials S1_Description of yoga program_08Jul2020.pdf]

Brief description of the yoga program

Each class will begin with Chanting. This will be followed by 10 mins of warm-up session which will include body scan and focus, pranayama/chanting, and joint mobilization. This will be followed by 10 mins of sun salutations. Next will be 10 mins each of variable standing asanas and floor asanas. This will be followed by 10 mins of savasana comprising, tense and release, progressive body scan, breath and mind relaxation. Classes will end with 5 mins of asanas comprising, introduce awareness and movement, side lying, return to sitting, chant and meditation.

Yoga pose	Duration
Centering and Chanting	5 mins

<u>Centering</u>

This includes a focus on postural alignment, breath awareness, and an inward focus. An intention is set to foster mindfulness and disregard distractions or concerns outside of yoga practice.

<u>Chanting</u>

The chant “Hari Om” is a Sanskrit phrase that is intended to raise vibrational energy and erase suffering. This can be thought of in a universal sense, but can also apply to individual practitioners who suffer from chronic pain.

Yoga pose	Duration
Warm-up	10 mins

- **Body scan and focus**
- **Pranayama**
- **Joint mobilization**

<u>Body Scan</u>

This practice fosters mindful observation of the state of the body and equanimity regarding the relationship to the body as it changes. It also ensures safety in yoga practice by paying attention to the body’s experience and sensation.

<u>Pranayama</u>

Pranayama is controlled breath. While there will be attention to coordination of breath and movement throughout the practice, this beginning pranayama focuses on deep abdominal breathing to reduce the stress response and calm the autonomic nervous system.

<u>Joint Mobilization</u>

This sequence briefly mobilizes all major synovial joints of the body to reduce stiffness and increase mobility in preparation for larger movements.

Yoga pose	Duration
Sun Salutations	10 mins

Description

Further details provided in Yoga for arthritis, Teacher’s Manual, available at ARTHRITIS.YOGA

The Sun Salutation sequence includes 10 poses that will each be taught individually in the first class. Fluid movement between these 10 poses makes up the Sun Salutation, which will be practiced on both sides with individualized modifications during every class. Students may use the wall, a chair, or other props to safely execute the sequence without discomfort. The Sun Salutations will take longer to execute in early classes, but will be reduced to 10 minutes later in the intervention as students become more facile with it.

Yoga pose	Duration
Standing Asanas*	10 mins

Warrior 2 (Class 1)

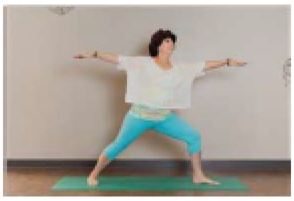

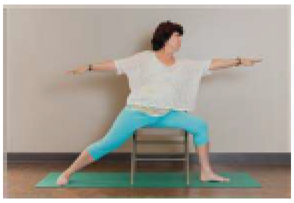

Directions

This is the first standing pose to be introduced, and feels awkward to many students at the beginning. There are a lot of postural considerations, so take your time experimenting with variations. The stance should be wider than most would imagine. After stepping wide on the mat, hold the arms straight out, with wrists and shoulders in one line. Look at the feet to ensure they are positioned under the palms. You may have to step wider to accomplish this, which will be important for knee alignment later in the pose. Toes on the front foot turn 90 degrees to face front, and toes on the back foot turn 45 degrees to face the corner. Many will not turn this back foot in enough, which will hinder the ability to orient the hips properly. If you have limitations in the ankle, due to fused bones, deformity, or joint pain, put a wedge or block under the back heel. Turn the head to gaze over the front shoulder, being sure not to lower the back arm. Bend the front knee. Be sure the knee does not go past the ankle. If it does, widen the stance. Shoulders and hips are square to the long edge of the mat, even though the gaze is to the front. Relax the shoulders away from the ears and lengthen the fingertips with energy reaching out to both sides. It is an option to perform this posture with the support of a chair.

Further details provided in Yoga for arthritis, Teacher’s Manual, available at ARTHRITIS.YOGA

Side Angle Pose (Class 5)

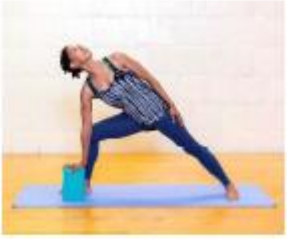

Directions

Starting in Warrior II, bring the front elbow to rest on the bent knee. Be sure that the knee is stacked over the ankle and not past. To go deeper, you might try lowering that hand to a block, keeping one shoulder stacked over the other. The other arm can extend alongside body, into cactus, or up alongside the ear. You can also bring the hand to rest on the upper hip. The goal is one continuous diagonal line from the back foot to the extended arm or crown of the head. You can gaze past the extended fingers, past the upper shoulder, or straight ahead. Watch for tension or condensing in the lower shoulder as you lean into the supporting elbow. The hips and shoulders should be in one plane, as though laying against a wall. Release any tension in the hand and fingers of the extended arm. Focus on a healthy alignment rather than trying to achieve a certain shape or depth in the pose.

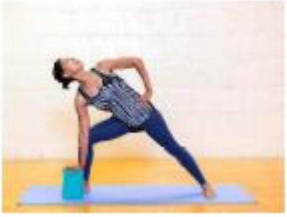

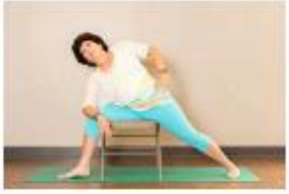

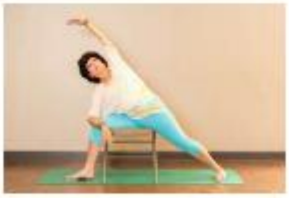

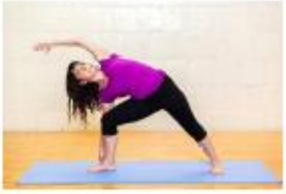

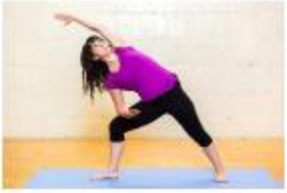

Reverse Warrior (Class 7)

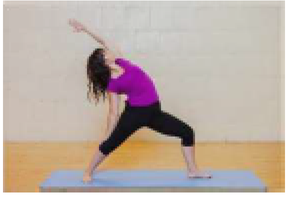

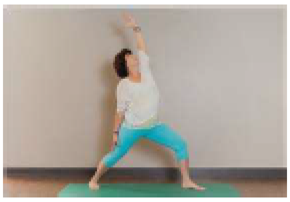

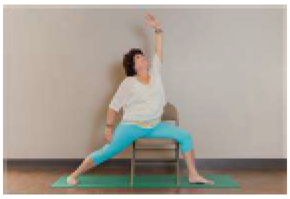

Directions

From the Warrior II stance, (either standing or seated) the back hand comes to rest on the outside of the back leg. It is not important how far down the leg this is. The hand can also rest on the back hip, if that is more comfortable. Avoid too much pressure on the back leg in order to protect the knee. The front arm reaches overhead in an arc, straight up, or in cactus. The front hand can also rest on the shoulder. Gaze is traditionally up into the palm, but take care to protect the neck. It is fine to look forward, if that feels better. Bend the knee deeply, as in Warrior I or II, being sure that the knee does not extend past the ankle and that weight is distributed evenly through the front foot. A wedge or other prop can be placed under the back heel or outside edge of the foot as needed.

Triangle (Class 3)

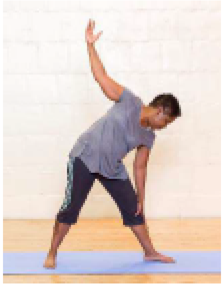

Directions

Begin in the same stance as Warrior II, with the front knee straight but not locked. Stand tall with arms extended to the sides and shift the torso forward toward the front leg, reaching forward with the front arm. From there, begin tilting the torso sideways so that the hand naturally reaches a resting place on your leg, a block, or a chair. Make sure you are not reaching too far down and compromising the alignment of the torso; keep one shoulder stacked over the other. Think about opening the front of that upper shoulder with the arm reaching toward the ceiling. The hand can also be placed on the upper hip with the elbow reaching upward. The gaze is traditionally up toward the open palm, but can be straight ahead or down for more comfort in the neck.

.

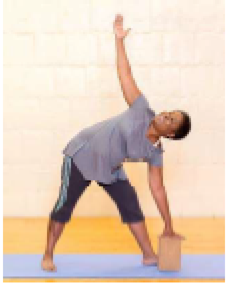

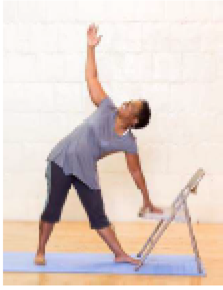

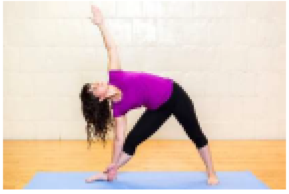

Tree (Class 1)

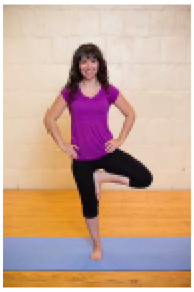

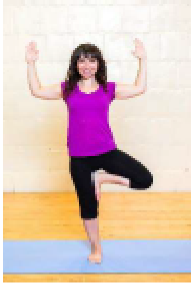

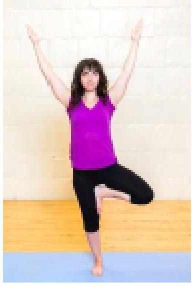

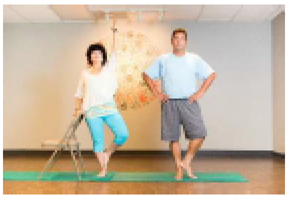

Directions

Standing on both feet, perhaps with the support of a chair or wall for balance, begin to shift the weight toward the left foot, coming to the ball of the foot on the right side. Rotate the right leg out from the hip and bring the right foot close to the left ankle. You can stay here, or try bringing the right foot to rest on the inside of the left ankle. Be sure toes point straight down, not forward. You might want to try sliding the right foot up to the left calf, or even using the right hand to bring the right foot up to the left thigh. Be sure to avoid putting the foot directly on the knee. The right leg acts like a buttress for a building to keep you steady, with pressure of foot and leg against each other creating resistance to maintain the pose. Keep length in the left side body without sinking into the left hip. Hands can be on the hips, at the heart, in cactus, or overhead. Keep shoulders relaxing down. If you are holding onto the foot, be sure not to hunch upper body forward, but to keep vertical spine. The gaze is up and out, on a focal point of your choice. Breathe deeply and repeat on the other side.

King Dancer (Class 3)

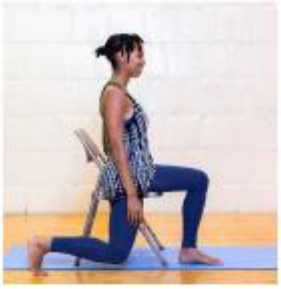

Directions

Most students will want to use a strap or chair for this pose. To use a strap, begin standing by the wall or a chair for support and wrap the strap around the front of the ankle, holding onto it with one hand or looping it over the same shoulder. Alternatively, place the foot or knee on a chair behind you. Hip/knee/ankle should be in one plane (so the knee is not torqued) and hips are square to the front. If feeling stable, the other arm can be raised overhead or to a cactus position. For greater challenge, the pose can be tipped forward with one line from fingertips to knee. There is a tendency for the angle of the hip to decrease or the back to round when extending forward. It is more important to keep a long spine than to tip forward. The supporting knee should be soft with weight evenly distributed on the whole foot.
A seated version of the pose begins in Seated Mounted with one knee lowering to the side of the chair. Be sure to avoid a pulling sensation in the front of the knee, so the stretch is felt more in the middle of the thigh and/or hip flexors.

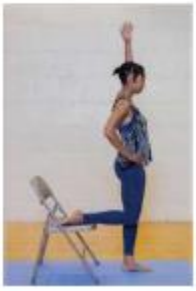

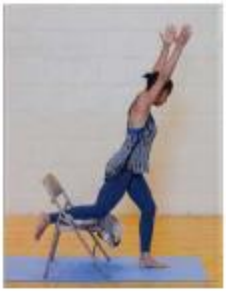

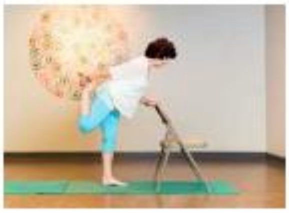

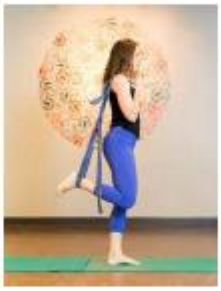

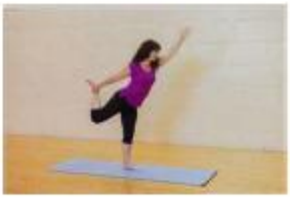

Further details provided in Yoga for arthritis, Teacher’s Manual, available at ARTHRITIS.YOGA

Warrior 3 (Class 5)

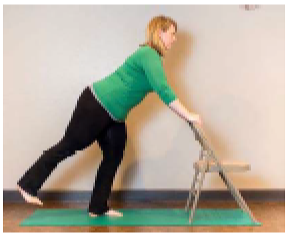

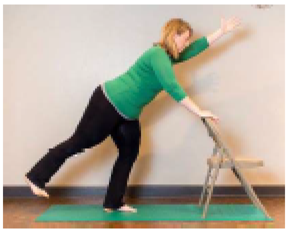

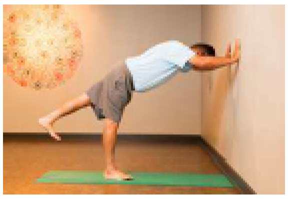

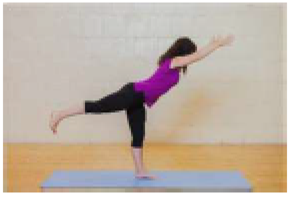

Directions

You may want to begin preparations for Warrior III at a chair or wall for support. This is a challenging pose, but it can be modified for broader accessibility. Begin by shifting weight into the left foot and extending the right foot back so that just the toes are touching the floor behind you. You can try lifting the right hand off of the chair/wall and extending it overhead or to cactus position. This may be your Warrior III. Consider a long line of energy extending from the left leg to the right arm and maintain the alignment of that line. If you like, you can begin lifting the left toes and tipping the torso forward. It is more important to maintain the line from fingertips to the heel of the extended leg than it is to tip forward. In fact, you might stay relatively upright, which is fine. Be sure that as you go deeper into the pose, the supporting knee remains soft with weight distributed through the whole foot. Gaze can be down or straight ahead. Gently return to Mountain and repeat on the other side.

.

Eagle (Class 7)

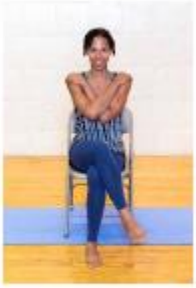

Directions

One knee crosses over the other, bringing them as close as possible, which is easier with a deep bend in knees and hips or seated in a chair. It can also be done against a wall for stability and support. For those with arthritis in hips, knees, ankles, or feet, it is not necessary to try bringing the foot around the back of the calf, as is done traditionally. It may be more comfortable to rest the foot on a block or other prop. The elbows cross in the opposite manner. It is fine if the elbows don't touch. More important is the idea of opposing twists. The lower body twists and crosses in one direction, while the upper body twists and crosses in the other direction, bringing the body back to center. Whether or not the palms are touching, fingers should be elongated and pointed upward, aiming to keep the eyes between the hands or the hands between the eyes.

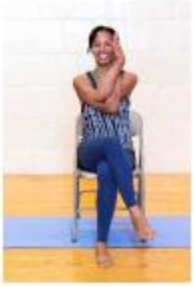

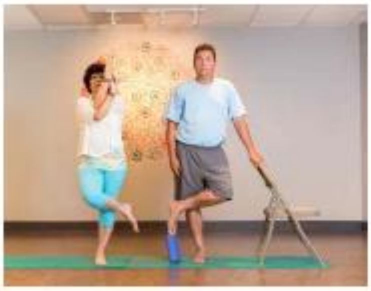

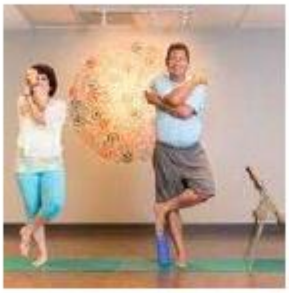

Goddess (Class 8)

Stand with the legs wide, as with Wide Angle Forward Bend. Place hands on the hips with the shoulders back and down. Rotate the legs outward from the hip so the toes are pointing slightly away from forward. Ensure adequate core support by engaging the abdominal muscles slightly while continuing to breath freely. Shoulders should be aligned directly over the hips with a lengthened spine. Bend the knees being sure to keep weight on the outside of the feet (without rolling in on the arch) and the knees bending directly over the feet. The bend can be very slight. Ensure that the knees do not bend past the toes. If there is sufficient stability, arms may be placed in "cactus" position with fingers spread wide apart and palms facing forward.

Half-Moon (Class 11)

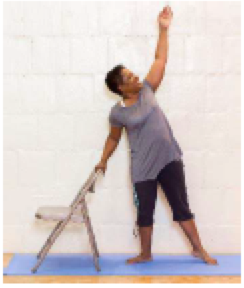

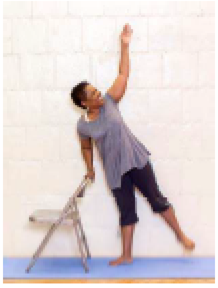

Directions

This is a fairly advanced pose and is not introduced until late in the session, perhaps moving from a standing pose or other balance. I recommend using a block (at any height) or a chair to support the pose without reaching for the floor. There is an intention toward creating one line from head to the heel of the extended leg. The other hand can be on the hip or reaching toward the ceiling within the limits of comfort for the shoulder. Gaze can be upward toward an extended arm, straight forward, or down toward the floor, depending on the comfort of the neck. This is best done against a wall at first, lying back against the wall to feel the expansiveness of the pose. It can then be replicated just an inch away from the wall to challenge balance, if desired.

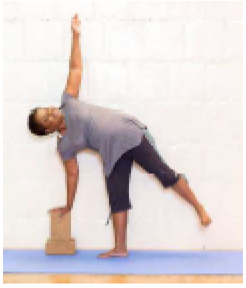

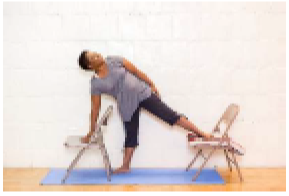

*All poses are highly adapted to individual needs and abilities using variations and props. Poses as executed in the intervention may not closely resemble traditional images or drawings.

Yoga pose	Duration
Floor Asanas*	10 mins

Head to Knee (Class 1)

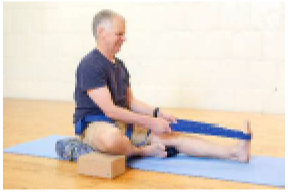

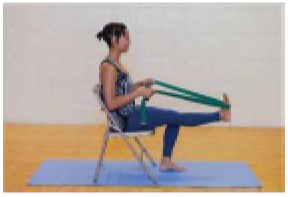

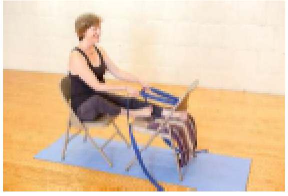

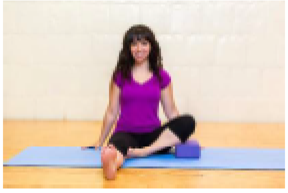

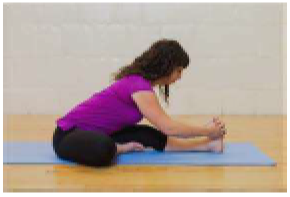

Directions

Starting in Staff pose on the floor or a chair, bend one knee toward the chest, planting the foot on the floor or chair. Let the knee open out to the side with the sole of the foot touching the inside of the opposite leg (like in Tree). You may want some support, like a block or blanket, under the knee. Knee and toes of the straight leg point up toward the ceiling. Sitting up tall here might be your full pose. If you have room to hinge forward, keep a long spine as you walk the hands down the sides of the extended leg. Work to center the sternum over the thigh so that the shoulders are somewhat even. Taking a look at the elbows can assist with this. They should be the same distance from the floor. A strap can be wrapped under the sole of the foot, holding onto the strap with both hands or looping the arms through the strap. Keep a long spine unless folding to 45 degrees, at which point you can release forward and surrender.

Seated Forward Fold (Class 5)

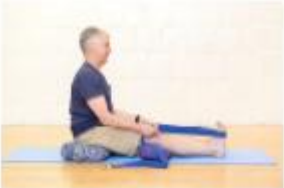

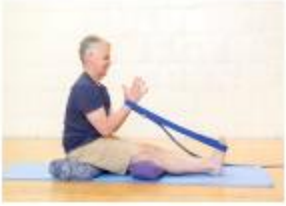

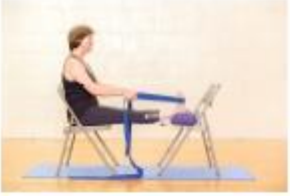

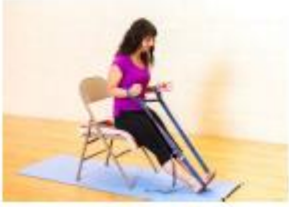

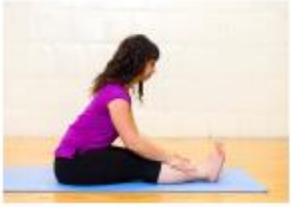

Directions

Start in Staff Pose, sitting up on a blanket or bending knees if the hamstrings are tight. Arms reach overhead and torso leans forward from the hips. A strap can be wrapped under the soles of the feet, holding on with both hands to pull further into the pose. If the hamstrings are loose and student does not have difficult grasping, the first two fingers can be wrapped around the big toe. Elbows open out to the side as the arms bend to draw the torso closer to the legs. For those with difficulty grasping, the arms can just rest on or beside the legs.

Tailor's Pose (Class 6)

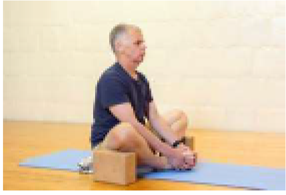

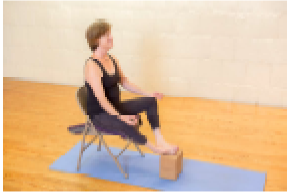

Directions

Knees are bent out to the sides, soles of the feet touching. Legs are pulled in as close to the body as possible, which may not be very far. Again, sitting up on a blanket or in a chair may help with being able to lengthen the spine. Keep the spine lengthened, bringing the sternum closer to the feet. Elbows can lower toward the knees, applying a bit of pressure to increase the stretch if available. If uncomfortable, blocks can be placed under the knees for support. Gaze is out past the feet, shoulders are relaxed away from the ears. If the torso moves past 45 degrees, you can allow the back to round slightly as you release forward into the pose. At this point, you may want to extend the arms forward or relax them to the sides.

Wide Angle Forward Bend (Class 11)

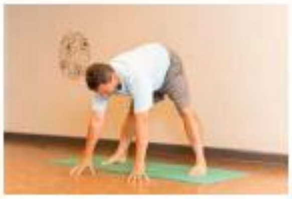

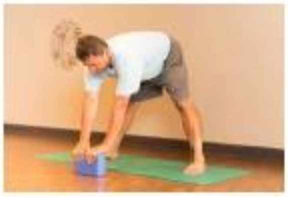

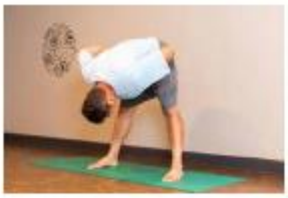

Directions

Start with legs as wide as they would be for Warrior II, but with both pointing straight ahead. Take your arms out to the sides for reference and consider bringing the feet as wide as the hands . Internally rotate the legs slightly (heels out, toes in). Hands are placed on the hips, with shoulder blades drawn together and down. Fold forward at the hips, trying not to lean back into the heels. If you feel unstable with the hands on the hips, they can be placed on floor, block, or chair.

Spinal Twist (Class 1)

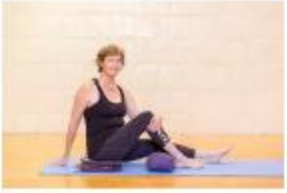

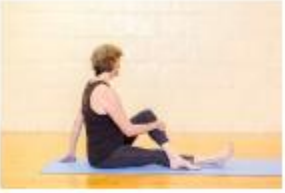

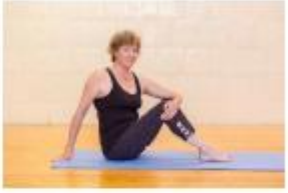

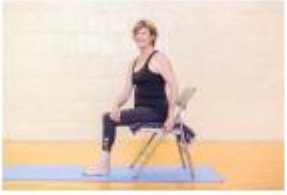

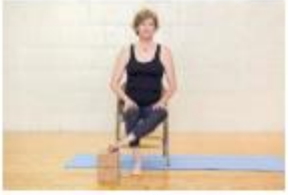

Directions

Start in Staff pose, either on the floor or in a chair, perhaps sitting on a prop (blanket, pillow, bolster). On the floor, pull one knee in as if preparing for Head-to-Knee pose. You might choose to leave the foot planted on the floor there, or lift and cross it over to the outside of the extended leg. In either case, imagine making a footprint on the floor. Drawing the foot in close to the body may help you sit up taller, as long as it feels okay for the hip and knee. In the floor version, wrap the opposite arm around the bent knee, hugging it toward the body as you sit tall and keep sitz bones planted evenly on the floor. In a chair, you may choose to keep the feet firmly planted or to cross one leg over the other. Sit up tall and begin to twist from the base of the spine toward the crossed leg, looking gently over shoulder.

Further details provided in Yoga for arthritis, Teacher’s Manual, available at ARTHRITIS.YOGA

Lying Extended Leg Pose (Class 4)

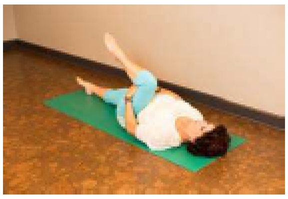

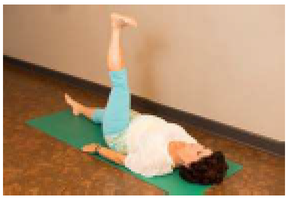

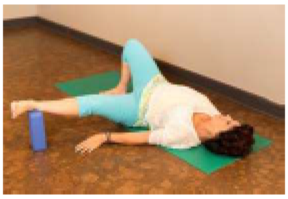

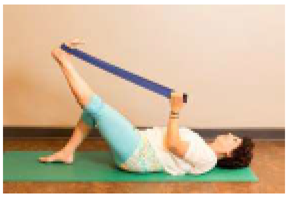

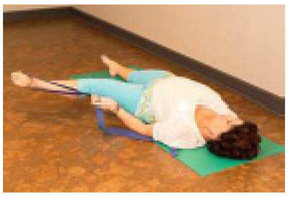

Directions

Lying on back, pull one leg into chest, holding behind the knee instead of on the shin. Allow the extended leg to relax on the floor. Alternatively, you could bring the sole of that foot to the floor for stability and ease in the low back, with the knee pointing up toward the ceiling. Most students will want to use a strap, wrapping it around the sole of the foot to extend the leg upward toward the ceiling. You can also hold the back of the leg as it extends. The head can be lifted briefly toward the knee, pulling in with the abdominal muscles. Continue by bringing both ends of the strap into one hand on the side of the lifted leg. The other hand should be placed on the hip of the lower leg or along side the body. The upper leg then opens out to the side, being sure to keep both hips on the floor. The leg will most likely not go to the floor, and that's ok because it can go to a block. If the hips are stable, the other arm can be extended out to the side, and possibly the head can turn in that direction. Switching the strap to the other hand, you can then move into an extended leg version of Lying Spinal Twist (see next page).

Lying Spinal Twist (Class 4)

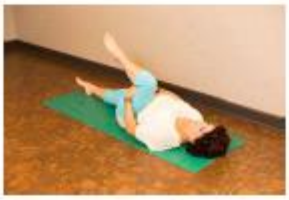

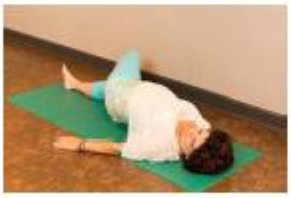

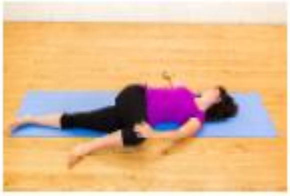

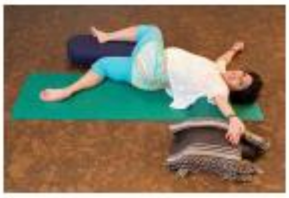

Directions

With our without a strap and moving from Extended Leg pose, the leg can be brought across the body to the floor on the other side. Other arm extends out to the side and the head turns in that direction. Although there is some active stretching of the hamstrings by holding the strap or leg, the spinal twist is passive. Leg should be relaxed on the floor as the opposite shoulder reaches toward the floor. It is fine if the shoulder doesn't touch, and the hip of the top leg should be off the floor so the twist extends through the whole spine. The spinal twist can also occur after Bridge, starting with both knees bent into the chest, dropping the knees over to one side. Both arms are extended out to the sides and the head turns away from the knees. In any spinal twist, if arthritis in the spine is hurting, the legs can rest on a block or bolster to decrease the intensity of the pose. If turning the head is bothersome, that step can be left out.

Bridge (Class 5)

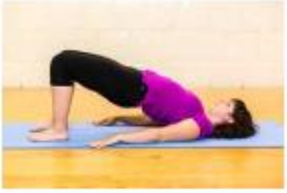

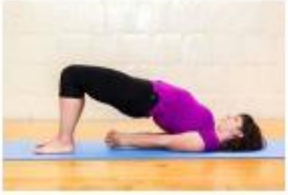

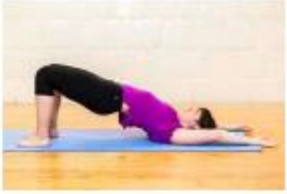

Directions

Lying on the back, soles of the feet are placed on the floor, about one foot from the hips. Feet should be hip distance apart and knees pointing straight upward. Arms are extended alongside the body. Pressing into the feet, hips are lifted off of the floor. You may not have enough strength to lift the hips at first, which is fine. Simply engage the muscles as if you were going to lift, and eventually you will build up enough strength for that to happen. There is as much benefit in doing this as there would be for someone who could lift the hips. It is important to continue breathing and not strain to accomplish the movement. There is a tendency for the knee positioning to be compromised, and for the angle of the feet to change as you move into the pose. Toes should point straight forward. You can also use a strap to keep the knees from opening outward. For more of a challenge (or when doing several rounds), you can try reaching the hands for the heels and/or lifting one leg at a time toward the ceiling. .

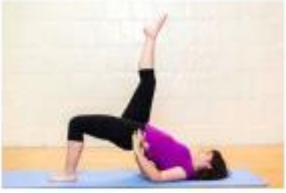

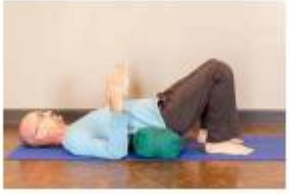

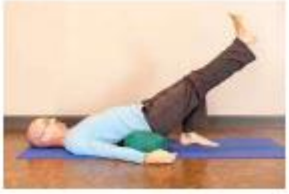

Locust/Bow (Class 8)

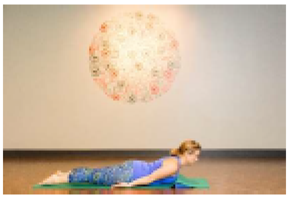

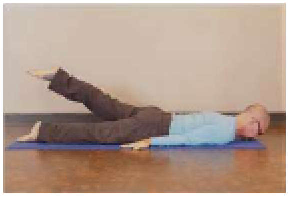

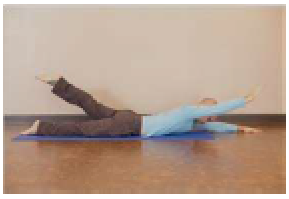

Directions

Begin lying on the abdomen, arms alongside the body. Rest the forehead on the floor or a folded blanket for a few breaths. Try rocking the body side to side, tucking the hands under the thighs, palms facing up. You may be able to tuck the elbows inside the hip bones but if neither of these positions is comfortable, the arms can be to the sides of the body. Having the palms face down may provide more stability. For half locust, lift one leg, keeping the hips level, forehead or chin can remain on the floor to support the cervical spine. Switch to the other leg. If you want to try full locust, bring both legs together and lift simultaneously. This is challenging and legs may not lift very high. Be sure to keep knees lengthened, since there is a tendency to bend the knees for greater height. Once again, it is not about the height of the pose, but the intention, muscle engagement, breath, and integrity of the alignment. Focus on engagement of the core muscles and be sure there is no aggravation of the low back or neck.

Child/Puppy (Class 8)

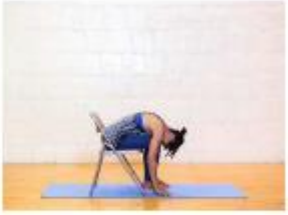

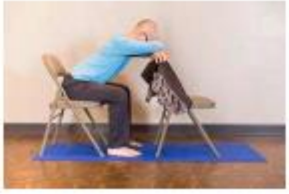

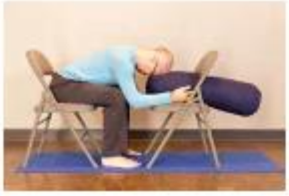

Directions

Child's pose is supposed to be grounding and restful, but that may not be true for those with arthritis in the knees or hips. A variety of variations are offered here to bring ease and grounding into the pose. You might start standing on knees, just like for Camel pose, perhaps with cushioning under the knees. Sit back on heels, a block between the feet, or a blanket. Bring forehead to the floor with arms extended out in front or alongside the body. If all of these poses are uncomfortable, hips can be raised to decrease knee angle. Alternatively, try sitting on a chair and folding forward to support the forehead with cushioning on another chair. Props can be used creatively to soften this pose, so experiment with options until you find what works for you.

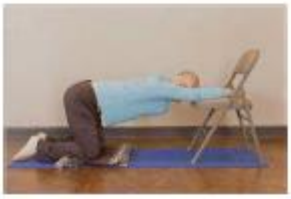

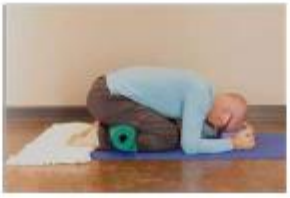

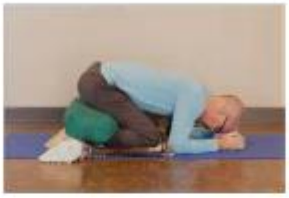

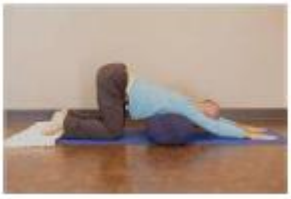

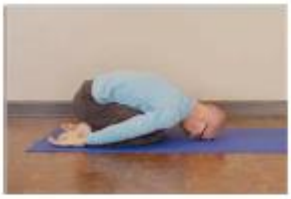

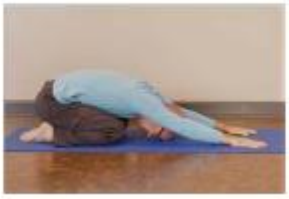

Further details provided in Yoga for arthritis, Teacher's Manual, available at ARTHRITIS.YOGA

Restorative Pose Options (Class 12)

Directions

WIDE ANGLE FORWARD BEND- Siting on the floor, perhaps on a prop, open the legs out to the sides. Place bolsters, blocks, or other props in front of you. Hinge at the hips and lower forward, relaxing the forehead or torso on those props. This can also be done seated in a chair, with another chair in front of you to rest on. Alternatively, you can use a standing version that isn't truly a restorative, but may still be grounding and relaxing. For that version, start with the feet wide and then pigeon-toe the feet slightly, bringing heels out and toes in, about 20 degrees. Bring hands to the hips and hinge forward, keeping a lengthened spine. Hands can be placed on a chair, block or the floor. Head hangs without tension in the neck or shoulders.

LYING SUPPORTED SPINAL TWIST- Lying on back with knees curled into chest or flat on the floor, open arms out to both sides. Allow knees to fall to one side while head turns to the other. Knees should rest on blankets and/or blocks for greatest comfort and ease. A blanket can also be placed under the opposite shoulder.

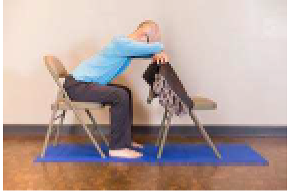

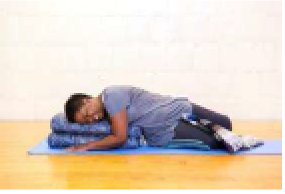

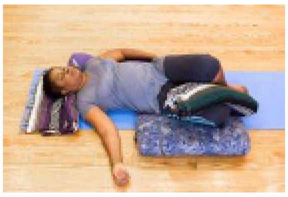

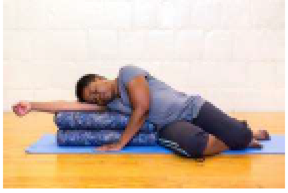

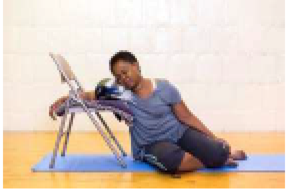

Further details provided in Yoga for arthritis, Teacher's Manual, available at ARTHRITIS.YOGA

Restorative continued

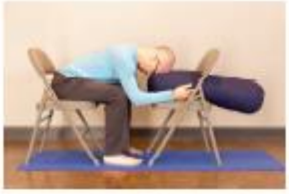

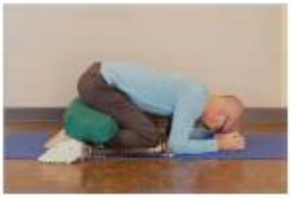

Directions

SUPPORTED CHILD- Standing on knees, with knees angled out and toes angled in, cushioning under the knees as needed. A blanket can be placed behind the knees to decrease the angle. You can also place a block or blanket between the legs to sit on, although some may find this uncomfortable. A stack of blankets should be in front of the body so that the upper body rests on that stack. The number of blankets necessary may vary considerably. It may be necessary to place blocks under the blankets for additional height. If none of these positions is suitable, hips can remain high while the upper body rests at a lower level.

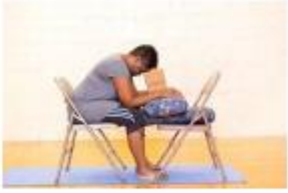

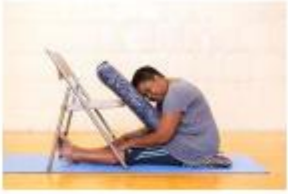

SUPPORTED FORWARD BEND- Sitting in Staff pose, on blankets if necessary for a vertical spine. Place a stack of blankets or bolsters across the thighs, high enough to rest arms and head. Lean over blankets resting chin on hands or turning head to one side. Alternatively, rest the forehead on a chair in front of you.

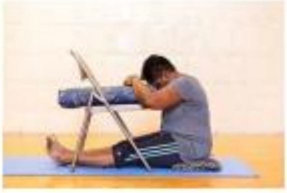

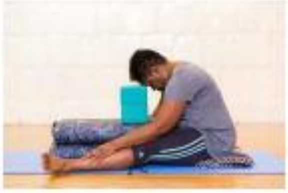

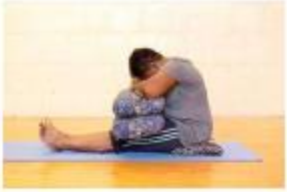

Restorative Continued

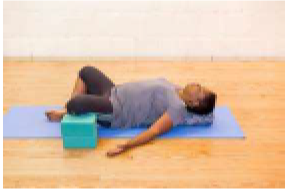

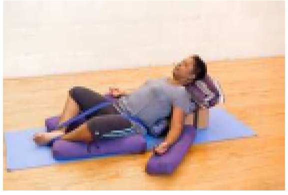

SUPPORTED RECLINING BOUND ANGLE- Sit in Tailor's pose with soles of the feet touching, knees supported as needed. You might try wrapping a strap around the outside of the feet, holding on to one side of the strap with each hand to slowly lower back. Lean back over a stack of blankets to recline. Blankets should be folded narrowly, so that the shoulders fall out to either side. You can continue holding onto the strap (with little muscle effort) for more of a stretch in the hips, or allow the arms to open out to the sides for more chest opening.

*All poses are highly adapted to individual needs and abilities using variations and props. Poses as executed in the intervention may not closely resemble traditional images or drawings.

Further details provided in Yoga for arthritis, Teacher's Manual, available at ARTHRITIS.YOGA

Yoga pose	Duration
Savasana	10 mins

- **Tense and Release**
- **Progressive Body Scan**
- **Breath and Mind relaxation**

Savasana

Savasana (Corpse pose) is traditionally done lying on the back without any propping. For a supported Corpse, you can experiment with what will be most comfortable, relaxing and sustainable for the duration of the progressive relaxation. Because Corpse lasts several minutes and the focus should be on restoration and relaxation, it is important to find a position without strain. You may want to put a rolled blanket under the knees, rest the lower legs on a chair, place a narrowly folded blanket along the spine, support the lumbar region, or place something under the head. These are a few examples but take your time and make adjustments until you feel satisfied with the position. Even then, it is okay to change position during Savasana if it is no longer comfortable or is hindering relaxation.

Yoga pose	Duration
Closing	5 mins

- **Introduce awareness and movement**
- **Side lying**
- **Return to sitting**
- **Chanting**
- **Meditation**

Chant

The closing chant will use the phrase "Om Shanti" which is a chant for peace. This can be thought of as universal peace or as individual peace within.

Meditation

Yoga programs often include brief periods of meditation before or after the asana practice. During these times, some instructors give guidance on how to approach meditation. During meditation one remains quiet and focus the mind, relieving it of the unnecessary clutter of trivial thoughts that stream in and out during the day. This discipline of the mind is said to provide relief from the day's stresses. Meditation can have any focus, such as the breath, an image, an idea or affirmation, a sound, or a personal prayer. In this intervention, we will allow each individual participant to choose a focus that feels comfortable and meaningful to them, with the goal of returning to that focus when noticing distractions.
