# Supplementary material for: A Randomised Controlled Trial of YOGa and Strengthening Exercise for Knee OsteoArthritis: Protocol for a Comparative Effectiveness Trial (YOGA Trial)
Source: J Funct Morphol Kinesiol. 2022 Oct 12;7(4):84. doi: 10.3390/jfmk7040084 (PMC9624302; doi:10.3390/jfmk7040084)
Supplement: Supplementary file 1 [file jfmk-07-00084-s001.zip › Supplementary Materials S2_Description Strengthening Exercises_19Jun2020.pdf]

Group strengthening program introduction

Group sessions will be held 3 times per week for 12 weeks. Each group session will be 60 minutes and will comprise:

1. Assessment – 5 mins
2. Warm up – 5 mins
3. Lower limb strengthening exercises run as a circuit class – 45 mins
4. Cool down – 5 mins

Information – At the start of the first session before commencing the program (and reinforced in subsequent sessions), the instructor should advise patients that some degree of knee discomfort or pain is to be expected with the exercises and that this generally reduces after the first few weeks, following which they should expect to see improvements in their knee pain and function. However, exercises should be performed within tolerable levels of pain. Any pain should also subside to usual levels by the next day with no increase in swelling following the exercise session. Patients should also be informed that when they start new exercises, there can be some soreness in the muscles which indicates that the muscles have been working. This is normal and will subside over a few days.

Assessment – At the start of each session, the instructor should ask the group how they found the previous exercise session and whether anyone had experienced any issues. This information can be used to modify the program for individuals.

Patient safety – This includes:

1. Asking patients to wear appropriate clothing to exercise
2. Bringing a drink bottle and towel
3. Letting the instructor know if they feel light-headed or dizzy during the exercises
4. Using hand support via a wall or chair for standing exercises if needed
5. Explaining that the exercises should be performed smoothly and slowly with quality of the movement emphasised.

Exercise dosage and progression

In the first week, the emphasis should be on learning the movements and using lower resistance. One set of exercises can be performed. The number of sets can be increased up to 2 and 3 over the weeks. After the first week, patients should be challenged by the exercises. During all exercises, their level of effort should be 5-7 out of 10 on the Modified Borg Rating of Perceived Exertion (RPE) scale shown on the following page.

Table: Modified Rating Perceived Exertion Scale (Day et al J Strength Conditioning Res 2004)

Rating	Descriptor
0	Rest
1	Very, Very Easy
2	Easy
3	Moderate
4	Somewhat Hard
5	Hard
6	-
7	Very Hard
8	-
9	-
10	Maximal

Progression of exercises is an essential component of the program and is determined by the instructor's assessment of the quality of the exercise performance and on the patient's RPE score for each exercise. A recommended progression rate is indicated for each exercise below. This is to be used as a guideline and the instructor may vary this rate of progression for an individual if they feel it is indicated. For example:

1. If the patient is not able to perform the task with good form after 3 weeks, they may remain on that level for another week.
2. If the patient is experiencing increased pain and swelling lasting more than one day after their exercises, they can go back to a previous level or do fewer repetitions of their current level.
3. If the patient is not sufficiently challenged by a level, they may be progressed earlier than indicated in the table below.

Table: Guide to progression for group strengthening exercise program

Level	Dosage	Progression (weeks)
**Warm Up**		
1. Hip Circles	Perform 20 circles in each direction. Switch legs. Progressively increase the size of the circles as you become more flexible.	-
2. Arm Circles	Gently perform 20 circles in each direction. Progressively increase the size of the circles as you become more flexible.	-
3. High Stepping	Perform "high-stepping" five times on each leg as you walk forward.	-
4. Heel-Toe Walk	Perform five times on each leg.	-
5. Marching on spot or walking around the room	Perform for 2 minutes	-
**Exercise 1. Forwards/ backwards**		
1. Sliding	1-3 sets of 10 with break of 30-60 seconds between sets	Weeks 1, 2 and 3
2. Sliding with Thera-band	2-3 sets of 10 with break of 30-60 seconds between sets	Weeks 4, 5 and 6

3. Stepping	2-3 sets of 10 with break of 30-60 seconds between sets	Weeks 7, 8 and 9
4. Stepping with Thera-band	2-3 sets of 10 with break of 30-60 seconds between sets	Weeks 10, 11 and 12.
**Exercise 2. Sideways exercise**		
1. Sliding	1-3 sets of 10 with break of 30-60 seconds between sets	Weeks 1, 2 and 3
2. Sliding with Thera-band	2-3 sets of 10 with break of 30-60 seconds between sets	Weeks 4, 5 and 6
3. Sliding with Thera-band and foam	2-3 sets of 10 with break of 30-60 seconds between sets	Weeks 7, 8 and 9
4. Sliding with Thera-band, foam and eyes closed	2-3 sets of 10 with break of 30-60 seconds between sets	Weeks 10, 11 and 12.
**Exercise 3. Hip muscle strengthening**		
1. Side leg raises in standing	1-3 sets of 10 with break of 30-60 seconds between sets	Weeks 1, 2 and 3
2. Crab walk	Total of 30 steps in each direction. [May do all 30 before changing direction or may do in smaller groups depending on available space]	Weeks 4, 5 and 6
3. Wall push	20 second holds with short break between efforts. Two sets of 5 with break of 30-60 seconds between sets.	Weeks 7, 8 and 9
4. Wall push with knee bending	Short break between efforts. Two sets of 5 with break of 30-60 seconds between sets.	Weeks 10, 11 and 12.
**Exercise 4. Knee muscle strengthening**		
1. Chair stands	1-3 sets of 10 with break of 30-60 seconds between sets	Weeks 1, 2 and 3
2. Chair stands with more weight on arthritis leg	2-3 sets of 10 with break of 30-60 seconds between sets	Weeks 4, 5 and 6
3. Wall squats	2-3 sets of 10 with break of 30-60 seconds between sets	Weeks 7, 8 and 9
4. Wall squats with more weight on arthritis leg / deeper angle	2-3 sets of 10 with break of 30-60 seconds between sets	Weeks 10, 11 and 12.
**Exercise 5. Step-ups**		
1. Step-ups	1-3 sets of 10 with break of 30-60 seconds between sets	Weeks 1, 2 and 3
2. Step-ups with 2kg weight	2-3 sets of 10 with break of 30-60 seconds between sets	Weeks 4, 5 and 6
3. Forward touch downs	2-3 sets of 10 with break of 30-60 seconds between sets	Weeks 7, 8 and 9
4. Forward touch downs with 2kg weight	2-3 sets of 10 with break of 30-60 seconds between sets	Weeks 10, 11 and 12.
**Exercise 6. Hamstring strengthening**		
1. Seated knee flexion	1-3 sets of 10 with break of 30-60 seconds between sets	Weeks 1, 2 and 3
2. Hamstring curls with resistance	2-3 sets of 10 with break of 30-60 seconds between sets	Weeks 4, 5 and 6

3. Hip Extension with knee straight	2-3 sets of 10 with break of 30-60 seconds between sets	Weeks 7, 8 and 9
4. Hip Extension with knee bent	2-3 sets of 10 with break of 30-60 seconds between sets	Weeks 10, 11 and 12.
**Exercise 7. Calf strengthening**		
1. Double calf raises	1-3 sets of 10 with break of 30-60 seconds between sets	Weeks 1, 2 and 3
2. Single calf raises	2-3 sets of 10 with break of 30-60 seconds between sets	Weeks 4, 5 and 6
3. Double calf raises from edge of step	2-3 sets of 10 with break of 30-60 seconds between sets	Weeks 7, 8 and 9
4. Single calf raises from edge of step	2-3 sets of 10 with break of 30-60 seconds between sets	Weeks 10, 11 and 12.
**Exercise 8. Seated Knee Extension**		
1. Seated knee extension with resistance	1-3 sets of 10 with break of 30-60 seconds between sets	Weeks 1- 12
**Cool Down**		
Hamstring stretch	3 x 30 second hold each leg	
Calf stretch	3 x 30 second hold each leg	
Quads stretch	3 x 30 second hold each leg	

<u>Warm up 5 mins</u>

1. Hip Circles

- Stand on one leg, using a wall or chair for support, and gently swing the opposite leg in circles out to the side. Perform 20 circles in each direction. Switch legs. Progressively increase the size of the circles as you become more flexible.

2. Arm Circles

- Stand with feet shoulder-width apart and hold arms out to the sides, palms down, at shoulder height. Gently perform 20 circles in each direction. Progressively increase the size of the circles as you become more flexible.

3. High-Stepping

- Stand with feet parallel to each other and at shoulder-width apart. Step forward with the left leg and raise the right knee high up toward your chest (use a wall for balance, if needed) and use both hands (or one, if using the other for balance) to pull the knee up farther. Pause and bring right leg back down; repeat with the other side and continue "high-stepping" five times on each leg as you walk forward.

4. Heel-to-Toe Walk

- Stand with feet shoulder-width apart and take a small step forward by placing the heel of the right foot on the ground and rolling forward onto the ball of your foot, rising as high as possible (as if standing on tip-toe), while bringing the left foot forward and stepping in the same heel-to-toe roll. Repeat five times on each leg.

5. Marching on spot or walking around the room

- 2 minutes

<u>Cool Down 5 mins</u>

Stretches – 3 x 30 second hold each leg

- Hamstring stretch
- Calf stretch
- Quads stretch

Exercise 1: Forwards/backwards exercise

Level 1: Sliding

<u>Starting position</u>:

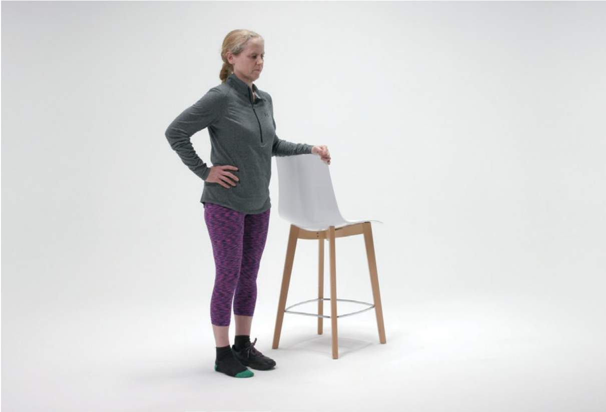

Standing on your arthritis leg with the non study leg on a sliding surface.

Sliding can be achieved by using a towel on smooth flooring or a plastic bag on the foot for carpet.

Use hand support for balance.

<u>Exercise</u>:

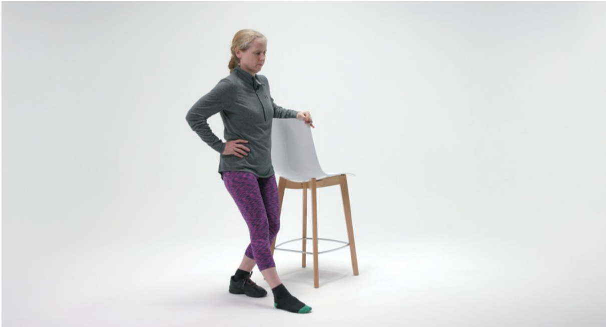

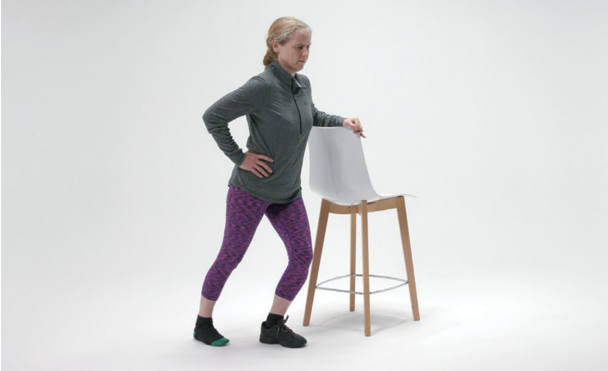

Slowly slide backwards and forwards with the 'sliding leg' while bending and straightening the arthritis leg.

Start with sliding just a few inches forwards and backwards and progress to larger slides as you gain control.

Keep your weight on the arthritis leg.

Concentrate on the alignment of your arthritis leg hip, knee and ankle – **position your knee over your foot** throughout.

Do 3 sets of 10 repetitions with a break of 30-60 seconds between sets.

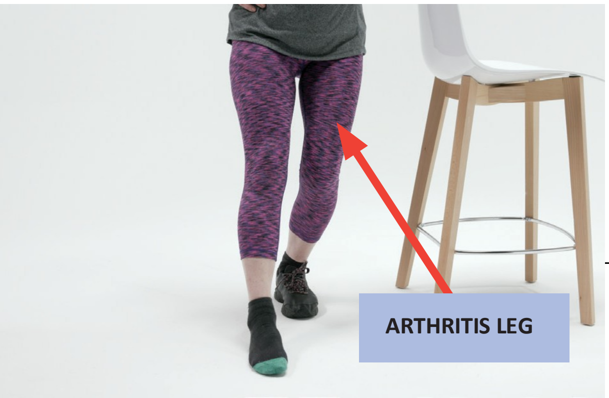

Exercise 1: Forwards/backwards exercise

Level 2: Sliding with Thera-band

Starting position:

Place a loop of Thera-Band around your arthritis knee and the leg of a table. This will provide a pull outwards on your knee that you must resist by aligning your knee over your foot through the whole exercise.

Your non study side foot should be on a sliding surface.

Sliding can be achieved by using a towel on smooth flooring or a plastic bag on the foot for carpet.

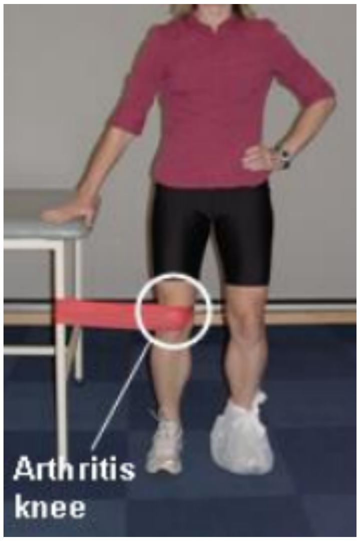

Exercise:

Slowly slide backwards and forwards with the 'sliding leg' while bending and straightening the arthritis leg.

Start with sliding just a few inches forwards and backwards and progress to larger slides as you gain control.

Keep your weight on the arthritis leg.

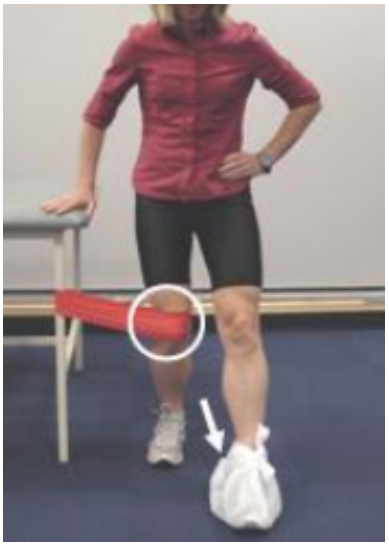

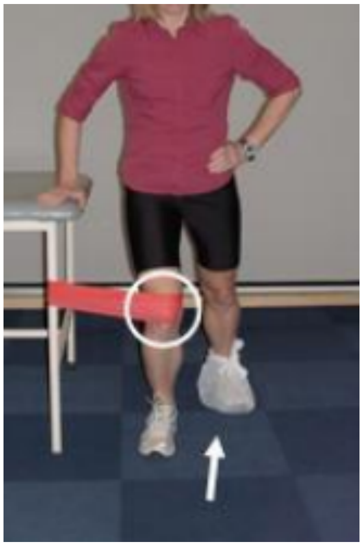

Concentrate on the alignment of your arthritis leg hip, knee and ankle – **position your knee over your foot** against the pull of the Thera-band throughout.

Do 3 sets of 10 repetitions with a break of 30-60 seconds between sets.

Exercise 1: Forwards/backwards exercise

Level 3: Stepping

Starting position:

Standing on your arthritis leg with your non-study leg behind.

Use a hand support for balance.

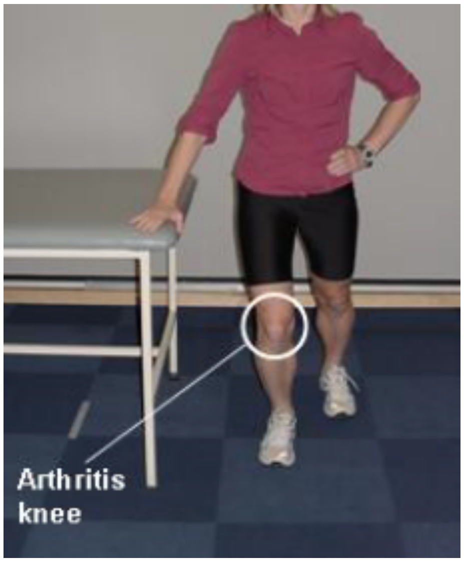

Exercise:

Instead of sliding forwards and backwards, **slowly** take a step forwards with your non-study leg to touch the floor. Then take a step backwards to the starting position.

Keep your arthritis knee slightly bent and your weight on your arthritis leg through the whole exercise.

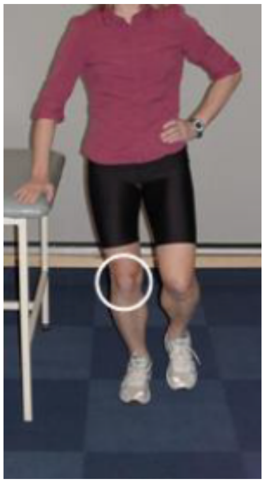

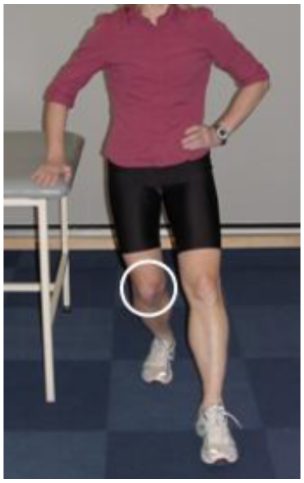

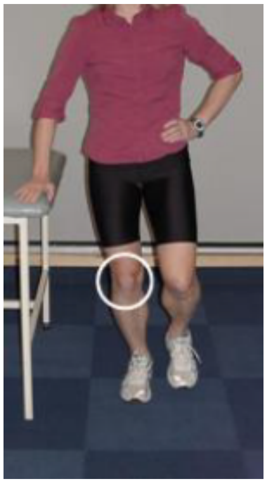

Start with a small step and progress to larger steps as you gain control.

Concentrate on the alignment of your arthritis leg hip, knee and ankle – **position your knee over your foot** throughout.

Do 3 sets of 10 repetitions with a break of 30-60 seconds between sets.

Exercise 1: Forwards/backwards exercise

Level 4: Stepping with Thera-band

Starting position:

Place a loop of Thera-Band around your arthritis knee and the leg of a table. This will provide a pull outwards on your knee that you must resist by aligning your knee over your foot through the whole exercise.

Start standing on your arthritis leg with non-study leg behind.

Use hand support for balance.

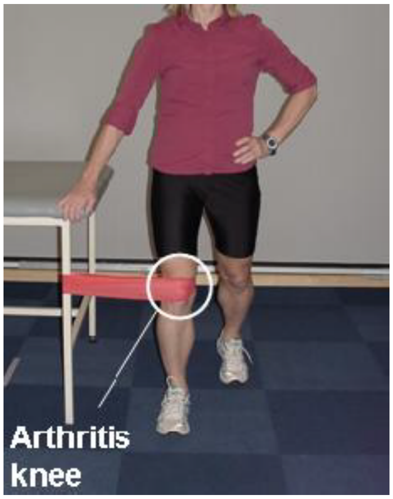

Exercise:

Slowly, take a step forwards with your non-study leg to touch the floor. Then take a step backwards to the starting position.

Keep your arthritis knee slightly bent and your weight on your arthritis leg through the whole exercise.

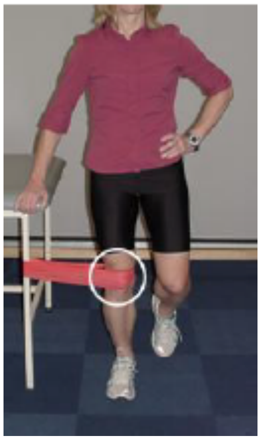

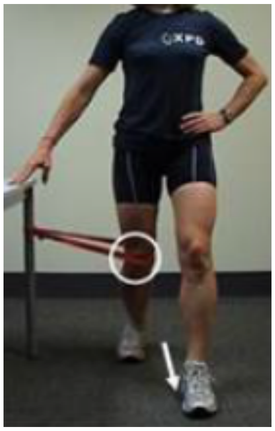

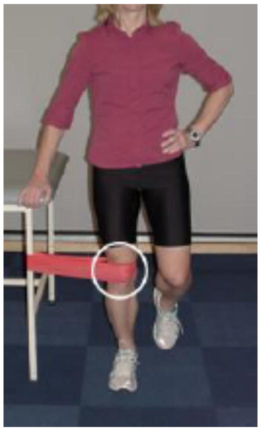

Start with a small step and progress to larger steps as you gain control.

Concentrate on the alignment of your arthritis leg hip, knee and ankle – **position your knee over your foot** against the pull of the Thera-band throughout.

Do 3 sets of 10 repetitions with a break of 30-60 seconds between sets.

Exercise 2: Sideways exercise

Level 1: Sliding

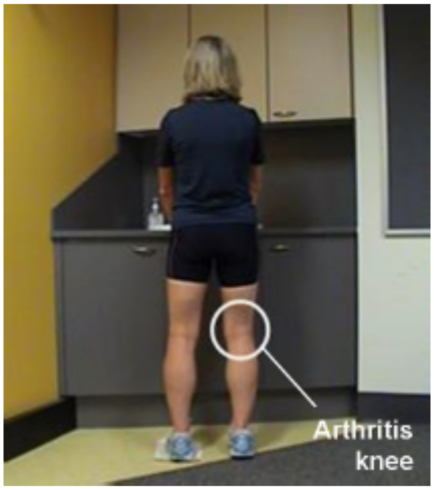

Starting position:

Standing on your arthritis leg with the other leg on a sliding surface.

Sliding can be achieved by using a towel on smooth flooring or a plastic bag on the foot for carpet.

Use hand support for balance.

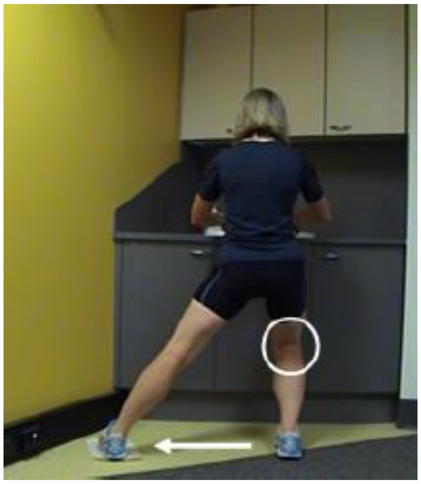

Exercise:

Slowly slide out sideways with the 'sliding leg' while bending the arthritis leg. Then slide back to the starting position.

Start with sliding just a few inches and progress to larger slides as you gain control.

Keep your weight on your arthritis leg through the whole exercise.

Concentrate on the alignment of your arthritis leg hip, knee and ankle – **position your knee over your foot** throughout.

Do 3 sets of 10 repetitions with a break of 30-60 seconds between sets.

Exercise 2: Sideways exercise

Level 2: Sliding with Thera-band

Starting position:

Place a loop of Thera-Band around your arthritis knee and the leg of a table. This will provide a pull outwards on your knee that you must resist by aligning your knee over your foot through the whole exercise.

Standing on your arthritis leg with the other leg on a sliding surface.

Sliding can be achieved by using a towel on smooth flooring or a plastic bag on the foot for carpet.

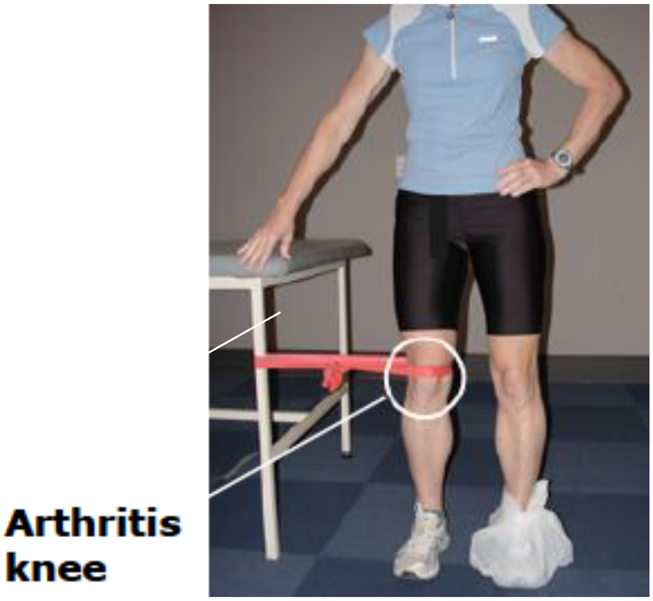

Exercise:

Slowly slide out sideways with the non-study leg while bending your arthritis knee.

Start with sliding just a few inches and progress to larger slides as you gain control.

Keep your weight on your arthritis leg through the whole exercise.

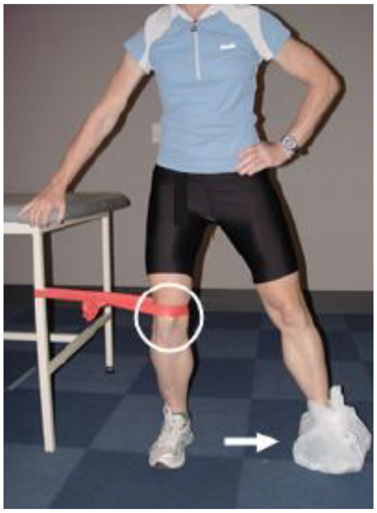

Concentrate on the alignment of your arthritis leg hip, knee and ankle – **position your knee over your foot** against the pull of the Thera-band throughout.

Do 3 sets of 10 repetitions with a break of 30-60 seconds between sets.

Exercise 2: Sideways exercise

Level 3: Sliding with Thera-band and foam

<u>Starting position</u>:

Place a loop of Thera-Band around your arthritis knee and the leg of a table. This will provide a pull outwards on your knee that you must resist by aligning your knee over your foot through the whole exercise.

Standing on your arthritis leg on your **foam cushion**, with your non-study leg on a sliding surface.

Sliding can be achieved by using a towel on smooth flooring or a plastic bag on the foot for carpet.

Arthritis knee

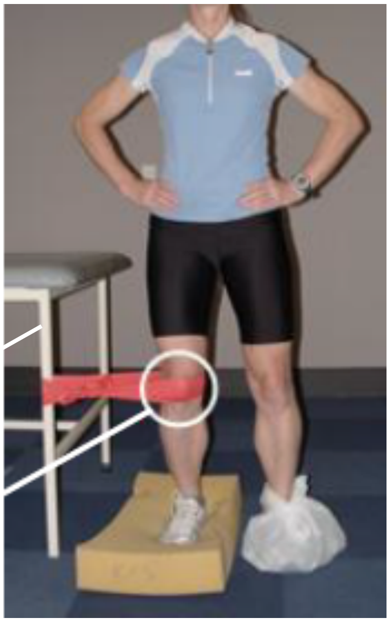

<u>Exercise</u>:

Slowly slide out sideways with the non-study leg while bending your arthritis knee.

Start with sliding just a few inches and progress to larger slides as you gain control.

Keep your weight on your arthritis leg through the whole exercise.

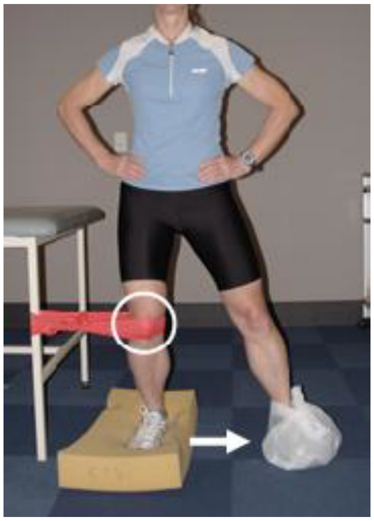

Concentrate on the alignment of your arthritis leg hip, knee and ankle – **position your knee over your foot** against the pull of the Thera-band throughout.

Do 3 sets of 10 repetitions with a break of 30-60 seconds between sets.

Exercise 2: Sideways exercise

Level 4: Sliding with Thera-band and foam and eyes closed

Starting position:

Place a loop of Thera-Band around your arthritis knee and the leg of a table. This will provide a pull outwards on your knee that you must resist by aligning your knee over your foot through the whole exercise.

Standing on your arthritis leg on your **foam cushion**, with your non-study leg on a sliding surface. Sliding can be achieved by using a towel on smooth flooring or a plastic bag on the foot for carpet.

Close your eyes.

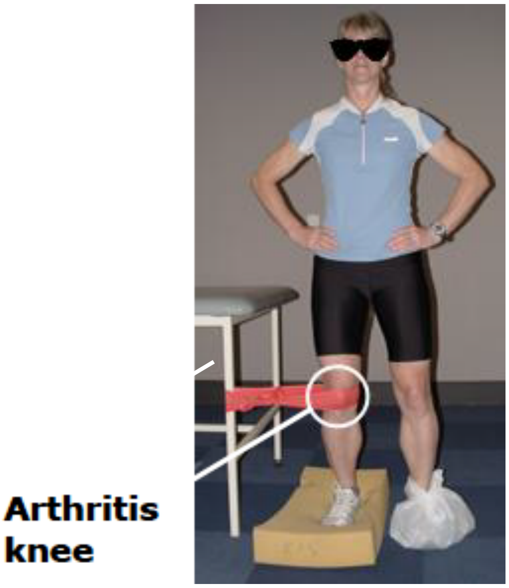

Exercise:

Slowly slide out sideways with the non-study leg while bending your arthritis knee. Start with sliding just a few inches and progress to larger slides as you gain control.

Keep your weight on your arthritis leg through the whole exercise.

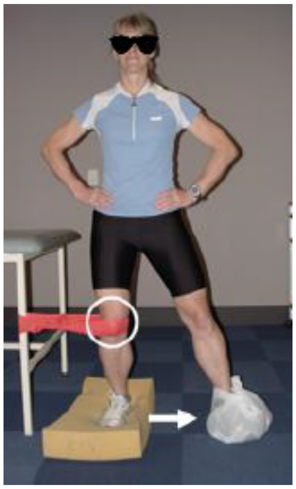

Concentrate on the alignment of your arthritis leg hip, knee and ankle – **position your knee over your foot** against the pull of the Thera-band throughout.

Do 3 sets of 10 repetitions with a break of 30-60 seconds between sets.

Exercise 3: Hip muscle strengthening

Level 1: Side leg raises in standing

Starting position:

Use the back of a chair or a wall to provide support.

Keep your back straight and facing forward. Don't twist as this will mean the wrong muscles are being exercised.

Loop your elastic band around your ankles.

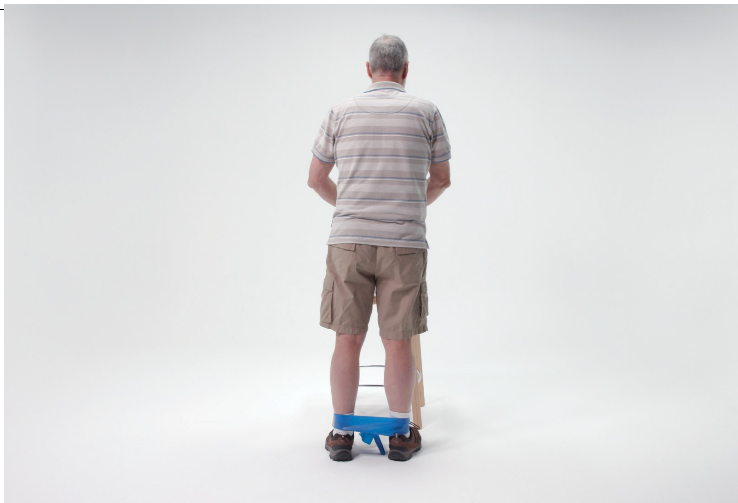

Exercise:

Keep your back straight. Try not to tilt to the side.

Keep your knee straight and your toes pointing forward.

Lift your arthritis leg out a small way to the side, leading with the heel.

Hold for 5 seconds and then lower slowly.

"Slowly out, hold, 2, 3, 4, 5, slowly in".

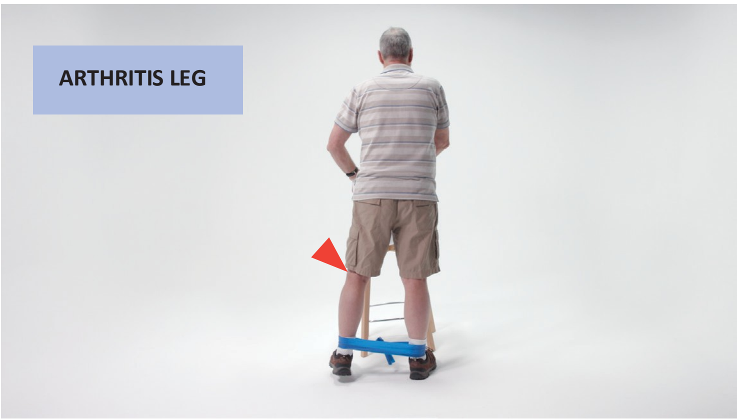

Exercise 3: Hip muscle strengthening

Level 2: Crab walking + red Thera-band

Starting position:

Place a loop of red Thera-Band around both ankles so that there is tension when ankles are separated 10cm. Slightly bend both knees.

For safety, you should stand facing a table, a kitchen bench or a wall which you can reach if you lose balance.

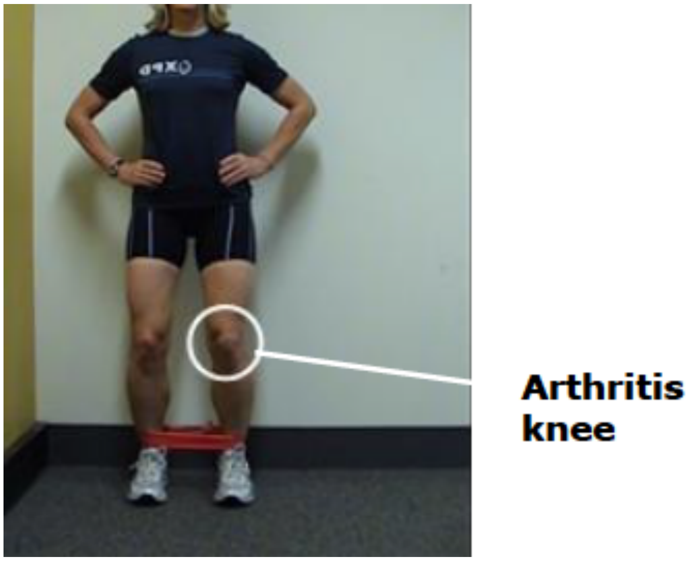

Exercise:

Step sideways against the pull of the Thera-band.

Do not twist or turn your body or legs. Your feet must point forwards while you are stepping sideways.

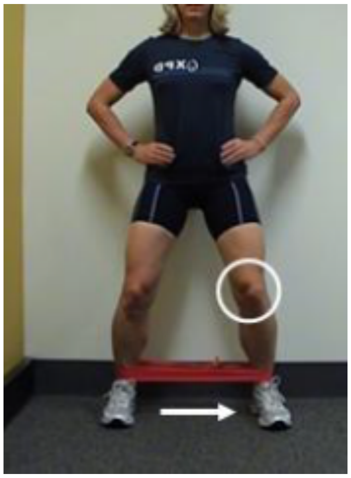

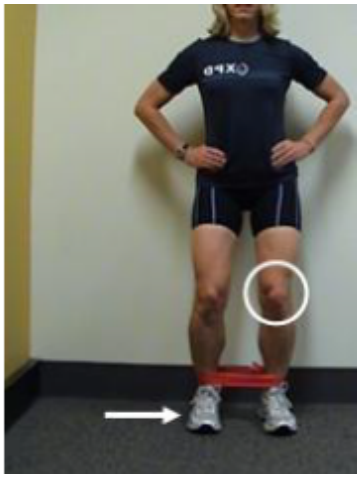

Concentrate on the alignment of both your arthritis and non-study legs – **position your knee over your foot** throughout.

Do a total of **30 steps in each direction**. For example, you can do all 30 in one direction around a table before changing direction. Or you can do 5 in one direction along a kitchen bench, change direction and do 5 back to the start and repeat this 6 times to reach your total of 30 in both directions.

Exercise 3: Hip muscle strengthening

Level 3: Wall push

Starting position:

Stand sideways to a wall with non-study leg against the wall.

Slightly bend arthritis knee to about 15-20°.

Lift the non-study leg just off the floor so that hip, thigh and knee are touching the wall.

Exercise:
Push your non-study leg into the wall and **hold for 20.** Return you foot to the floor and rest for a few seconds.

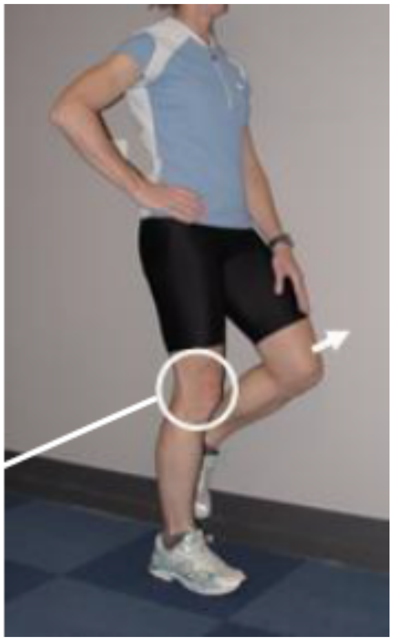

Arthritis knee

Concentrate on the alignment of your arthritis leg hip, knee and ankle – **position your knee over your foot** throughout.

Do 2 sets of 5 repetitions with a break of 30-60 seconds between sets.

Exercise 3: Hip muscle strengthening

Level 4: Wall push with deeper knee bending

Starting position:

Stand sideways to a wall with non-study leg against the wall.

Slightly bend arthritis knee to about 15-20°.

Lift the non-study leg just off the floor so that hip, thigh and knee are touching the wall.

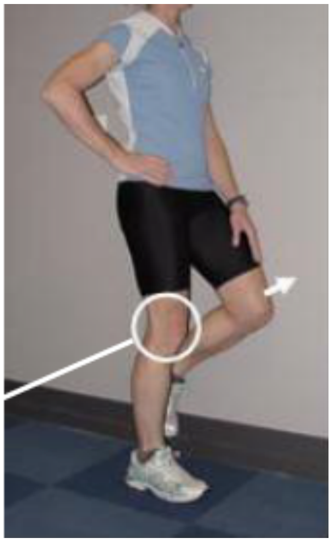

Arthritis knee

Activity:

Push non-study leg into the wall.

While continuing to push into the wall, slowly bend your arthritis knee to a maximum of 45°.

Straighten your knee and return your foot to the floor and rest for a few seconds.

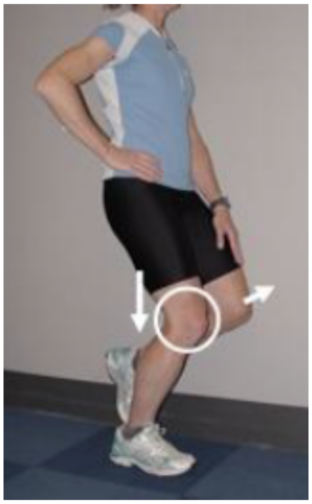

Concentrate on the alignment of your arthritis leg hip, knee and ankle – **position your knee over your foot** throughout.

Do 2 sets of 5 repetitions with a break of 30-60 seconds between sets.

Exercise 4: Knee muscle strengthening

Level 1: Chair stands

Starting position:

Sit on a standard height (eg. kitchen) chair, with your feet parallel and hip width apart.

Reach your hands out in front.

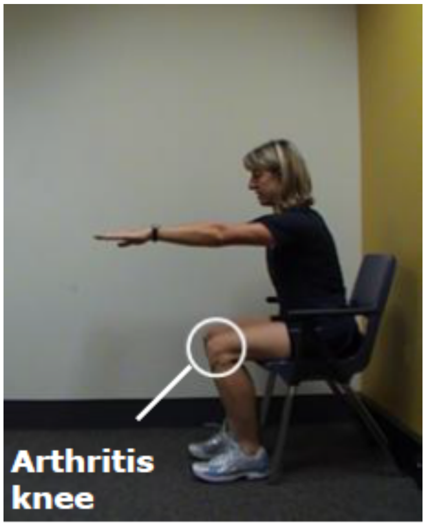

Exercise:

Stand up from the chair slowly (count 4 seconds) without using your hands.

Slowly return to sitting (count 4 seconds).

"Up, 2, 3, 4, down, 2, 3, 4"

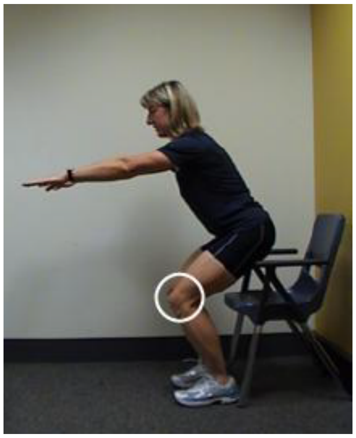

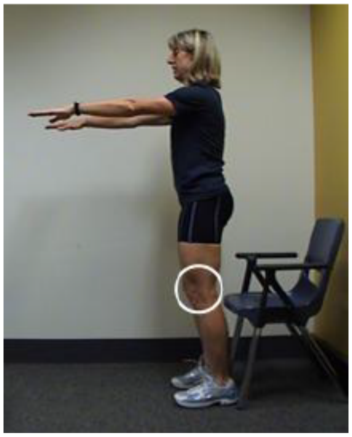

Concentrate on the alignment of both your arthritis leg and your non-study leg – **position your knee over your foot** throughout.

Do 3 sets of 10 repetitions with a break of 30-60 seconds between sets.

Exercise 4: Knee muscle strengthening

Level 2: Chair stands with more weight on arthritis leg

Starting position:

Sit on a standard height (eg. kitchen) chair, with your feet hip width apart.

Take **more weight on your arthritis leg** by either (a) placing your non-study leg further forward, or (b) shifting your feet sideways so your arthritis leg is in front of your body.

Reach your hands out in front.

(a)

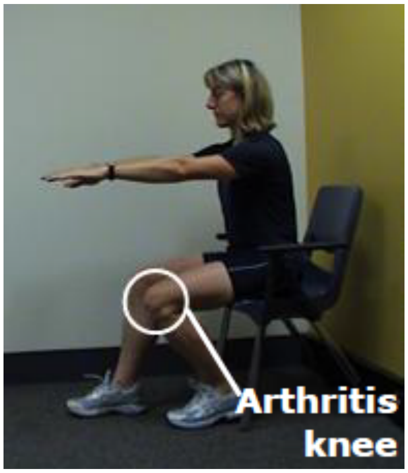

(b)

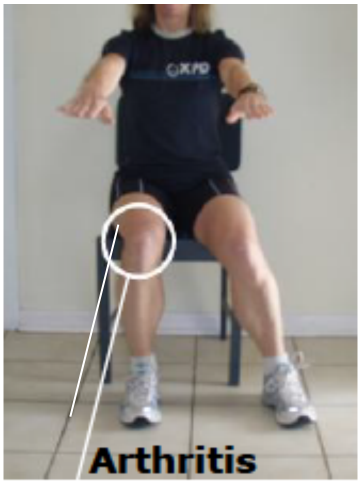

Arthritis knee

Exercise:

Stand up from the chair slowly (count 4 seconds) without using your hands.

Slowly return to sitting (count 4 seconds).

"Up, 2, 3, 4, down, 2, 3, 4"

Concentrate on the alignment of your arthritis leg hip, knee and ankle – **position your knee over your foot** throughout.
You **must** have **more weight on your arthritis leg through the whole exercise.**

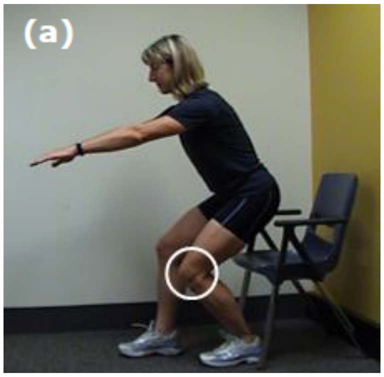

(b)

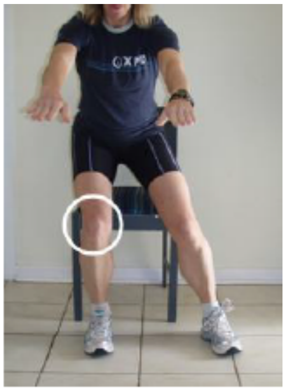

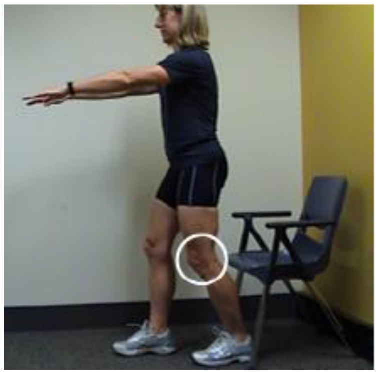

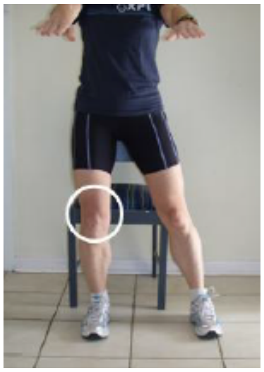

Do 3 sets of 10 repetitions with a break of 30-60 seconds between sets.

Exercise 4: Knee muscle strengthening

Level 3: Wall squats

<u>Starting position</u>:

Stand with your back to a wall, feet 10cm apart and 15cm away from the wall.

You may like a towel behind your hips to help you slide down the wall.

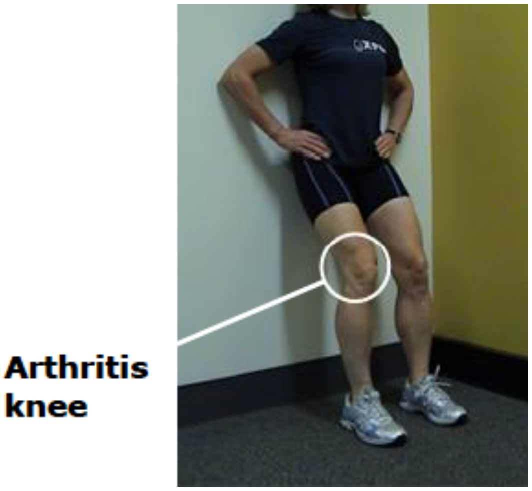

<u>Exercise</u>:

Slide **slowly** down the wall until your knees are bent about 30°, then slowly slide up again.

"Down, 2, 3, hold, 2, 3, up, 2, 3"

Your knees should stay in line with your feet.

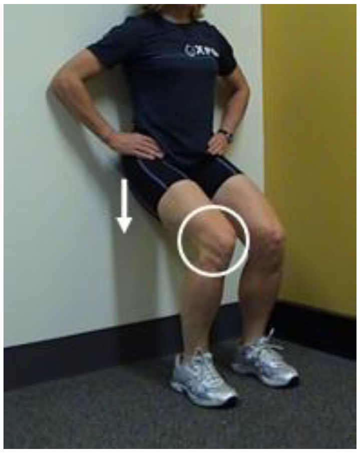

Concentrate on the alignment of both your arthritis and non-study legs – **position your knee over your foot** throughout.

Do 3 sets of 10 repetitions with a break of 30-60 seconds between sets.

Exercise 4: Knee muscle strengthening

Level 4: Wall squats with more weight on arthritis leg

Starting position:

(a) (b)

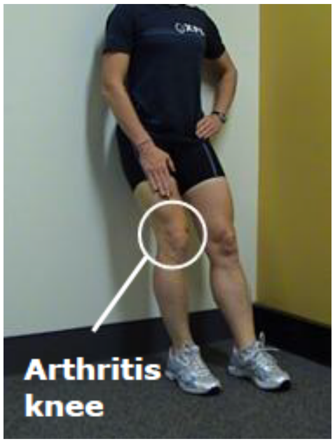

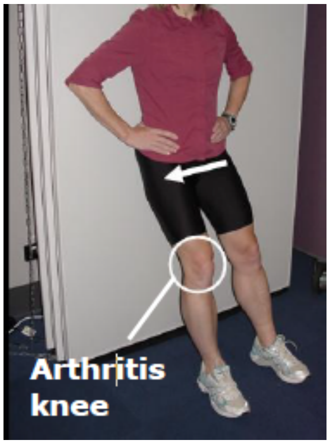

Stand with your back to a wall, feet 10cm apart and arthritis leg 15cm away from the wall. Your non-study side can be (a) further forward or (b) level with the arthritis leg but with your body shifted over the arthritis leg. You **must** have **more weight on your arthritis leg through the whole exercise**.

You may like a towel behind your hips to help you slide down the wall.

Exercises:

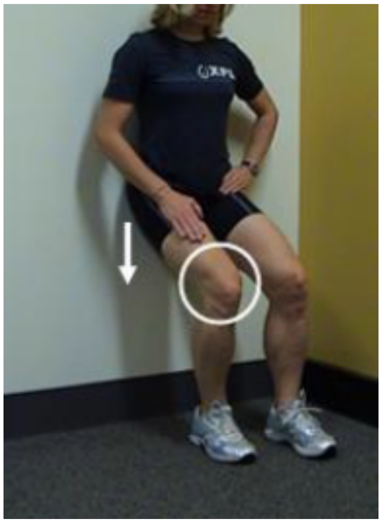

Slide **slowly** down the wall until your arthritis knee is bent about 30°, then slowly slide up again.

"Down, 2, 3, hold, 2, 3, up, 2, 3"

Your knees should stay in line with your feet.

You may use some scales under arthritis leg to check you keep more weight on that side.

Concentrate on the alignment of your arthritis leg hip, knee and ankle – **position your knee over your foot** throughout.

Do 3 sets of 10 repetitions with a break of 30-60 seconds between sets.

Exercise 5: Step-ups

Level 1: Step-ups

Starting position:

Place your arthritis leg onto a step in front of you.

Use a hand support (back of chair or handrail) for balance.

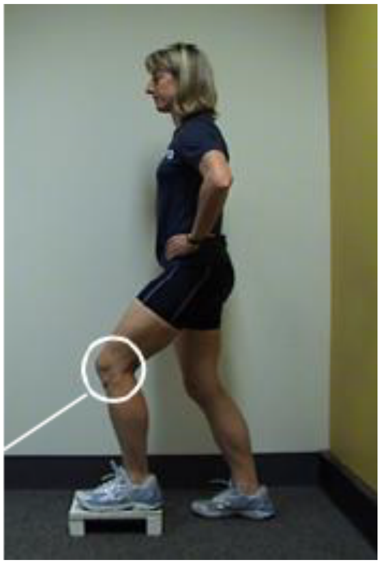

Arthritis knee

Exercise:

Step up onto the step slowly, carefully controlling the movement of your arthritis knee.

Just lightly touch your non-study leg to the step, and then step it back down slowly to the start position.

Your weight should be on your arthritis leg through the whole exercise.

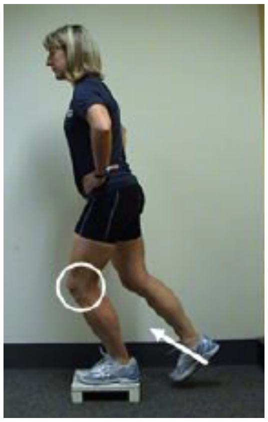

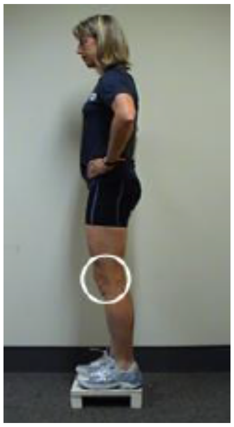

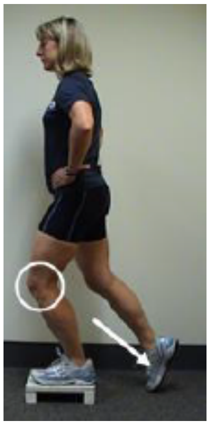

Concentrate on the alignment of your arthritis leg hip, knee and ankle – **position your knee over your foot** throughout.

Do 3 sets of 10 repetitions with a break of 30-60 seconds between sets.

Exercise 5: Step-ups

Level 2: Step-ups with weight

Starting position:

Hold 2kg of weight (a) against your chest, (b) in each hand, (c) in one hand while holding on for balance with the other, or (d) in a backpack.

(a)

Arthritis knee

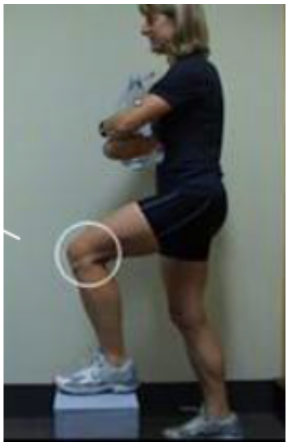

(b)

Arthritis knee

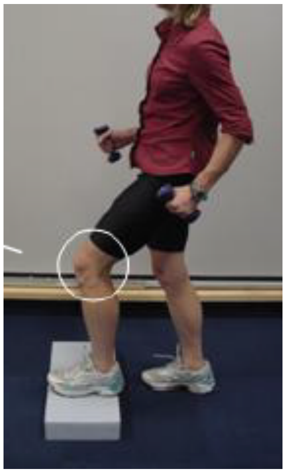

Place your arthritis leg onto a step in front of you.
Weight can be a 2L milk bottle filled (2kg) or half filled (1kg) with water.

Exercise:

Step up onto the step slowly, carefully controlling the movement of your arthritis knee.

Just lightly touch your non-study leg to the step, and then

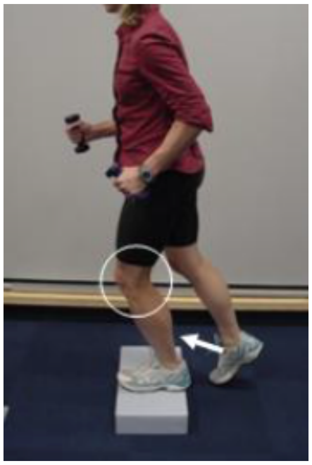

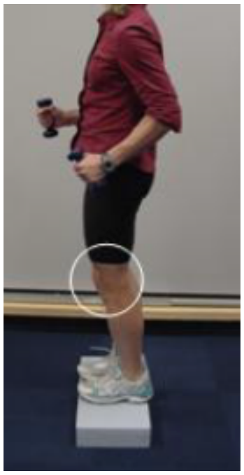

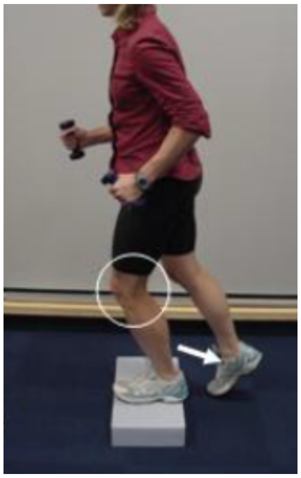

step it back down slowly to the start position.

Your weight should be on your arthritis leg through the whole exercise.
Concentrate on the alignment of your arthritis leg hip, knee and ankle – **position your knee over your foot** throughout.
Do 3 sets of 10 repetitions with a break of 30-60 seconds between sets.

Exercise 5: Step-ups

Level 3: Forward touch downs

Starting position:

Stand on the step.

Use a hand support (back of chair or handrail) for balance.

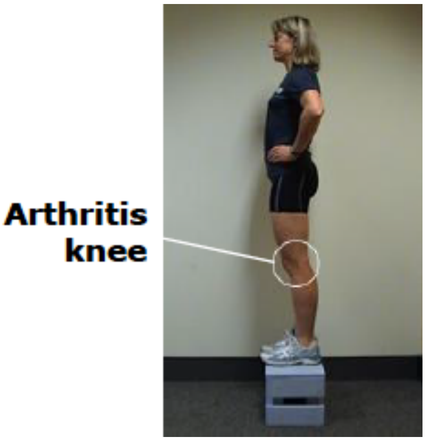

Exercise:

Controlling the movement of your arthritis knee, reach your non-study side towards the floor in front. If you can reach the floor, just touch it lightly. Return to the starting position.

Your weight should be on your arthritis leg through the whole exercise.

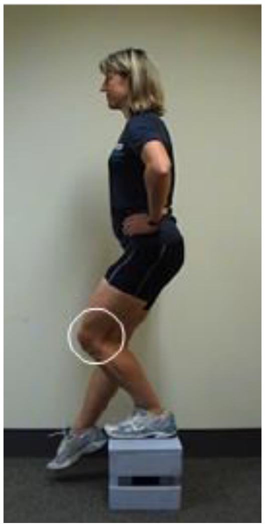

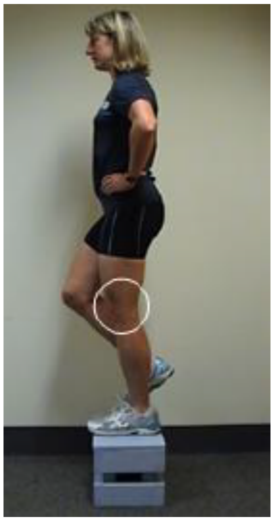

Concentrate on the alignment of your arthritis leg hip, knee and ankle – **position your knee over your foot** throughout.

Do 3 sets of 10 repetitions with a break of 30-60 seconds between sets.

Exercise 5: Step-ups

Level 4: Forward touch downs with weight

Starting position:

Stand on the step.

Hold 2kg of weight (a) against your chest, (b) in each hand, (c) in one hand while holding on for balance with the other, or (d) in a backpack.

Weight can be a 2L milk bottle filled (2kg) or half filled (1kg) with water.

(b)

Arthritis knee

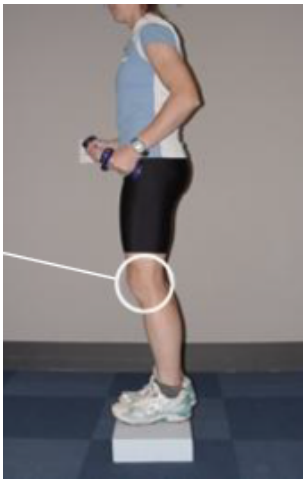

Exercise:

Controlling the movement of your arthritis knee, reach your non-study side towards the floor in front. If you can reach the floor, just touch it lightly. Return to the starting position.

Your weight should be on your arthritis leg through the whole exercise.

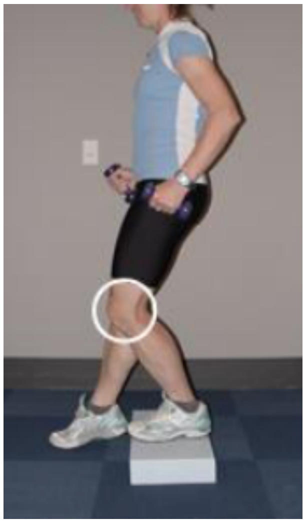

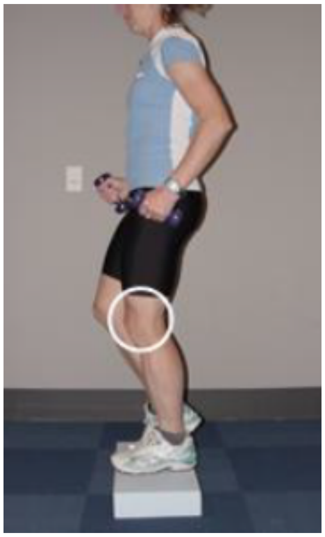

Concentrate on the alignment of your arthritis leg hip, knee and ankle – **position your knee over your foot** throughout.

Do 3 sets of 10 repetitions with a break of 30-60 seconds between sets.

Exercise 6: Hamstring strengthening

Level 1: Seated knee flexion

Starting position:

Sit upright in a chair. Move forward so you are sitting toward the front of the chair (so you have enough room to bend your knee under the chair).
Place one end of an elastic band securely around a stable object (e.g. a heavy table leg). Loop the other end around the ankle of your arthritis leg.

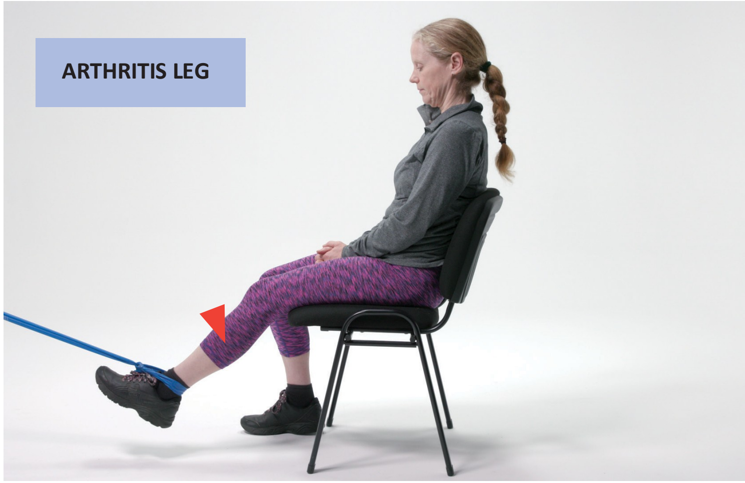

Exercise:

Keeping your opposite foot on the floor, pull against the elastic band and bend your knee more.

Your foot should move back further under the chair.

"Bend, hold, 2, 3, 4, 5, return".

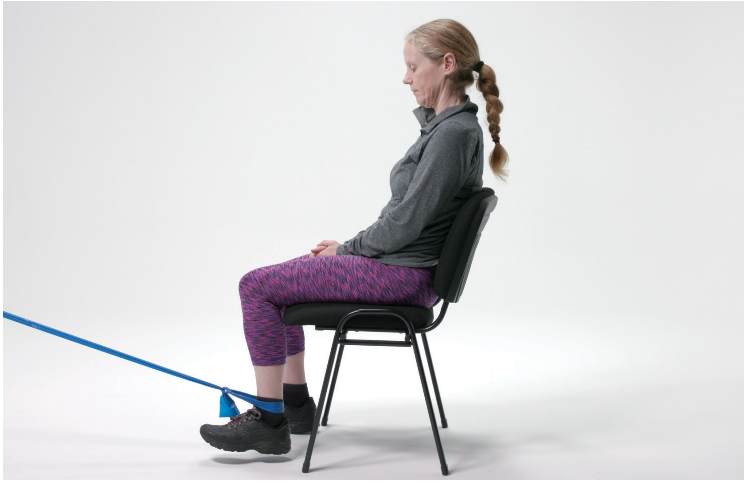

Do 3 sets of 10 repetitions with a break of 30-60 seconds between sets.

Exercise 6: Hamstring strengthening

Level 2: Hamstring curls with resistance

Starting position:

Place one end of an elastic band securely around the ankle of your arthritis leg.
Place the other end of the elastic around your opposite foot so you are standing on it.
Stand and lean forward on your forearms over a table.

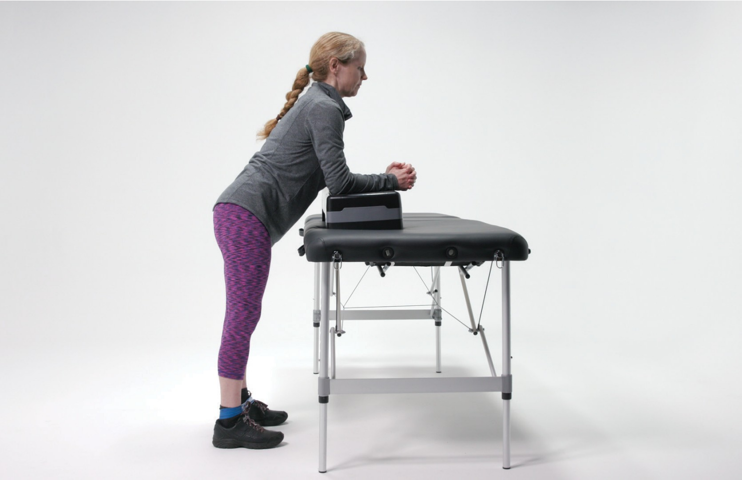

Exercise:

Slowly bend your arthritis knee
so that your heel comes up toward your bottom.

Hold for 5 seconds and lower slowly.
"Slowly up, hold, 2, 3, 4, 5, slowly down".

Do 3 sets of 10 repetitions with a break of 30-60 seconds between sets.

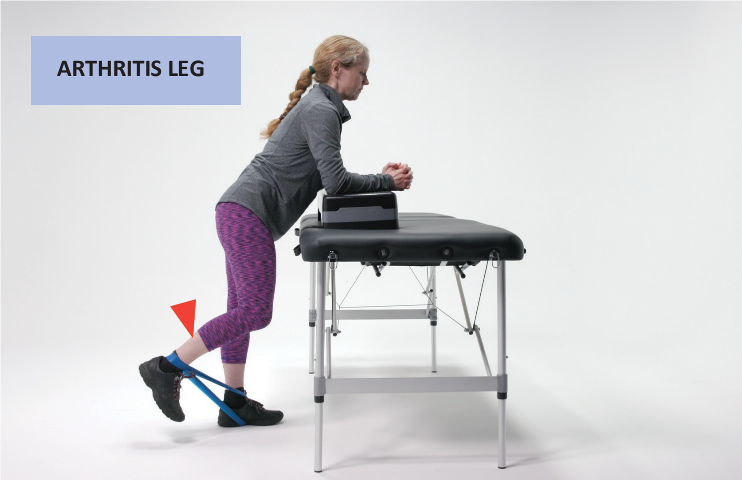

Exercise 6: Hamstring strengthening

Level 3: Hip Extension with knee straight

Starting position:

Place one end of an elastic band securely around the ankle of your arthritis leg. Place the other end of the elastic around your opposite foot so you are standing on it. Stand and lean forward on your forearms over a table.

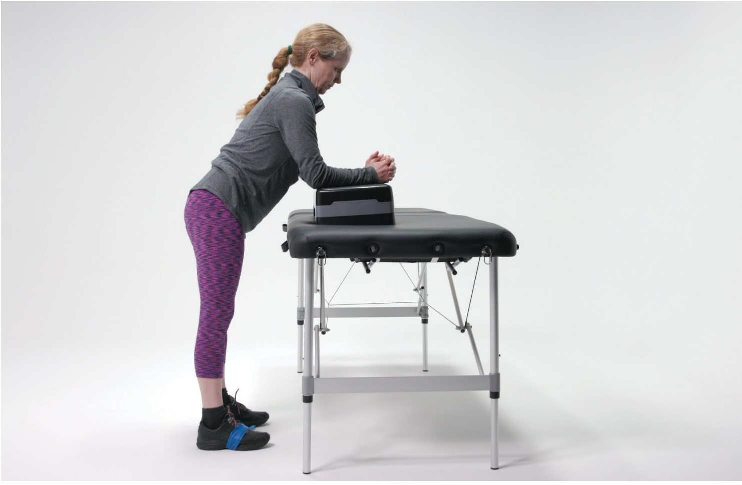

Exercise:

Lift your arthritis leg backwards behind you, keeping your knee straight. Lead with your heel, so you are extending at your hip joint. Keep the leg straight.

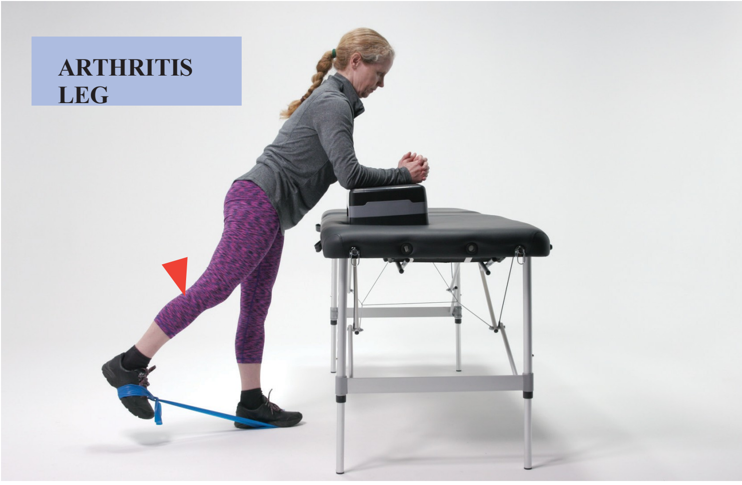

Hold for 5 seconds then slowly return to the starting position.

"Move behind, hold, 2, 3, 4, 5, slowly return".
Keep both legs straight.

Keep your hips facing forwards.

Don't twist your hips. Be careful not to over arch your back.
If you feel discomfort in your lower back don't move the leg back as far.

Do 3 sets of 10 repetitions with a break of 30-60 seconds between sets.

Exercise 6: Hamstring strengthening

Level 4: Hip extension knee bent

Starting position:

Stand and lean forward on forearms over a table or bench.

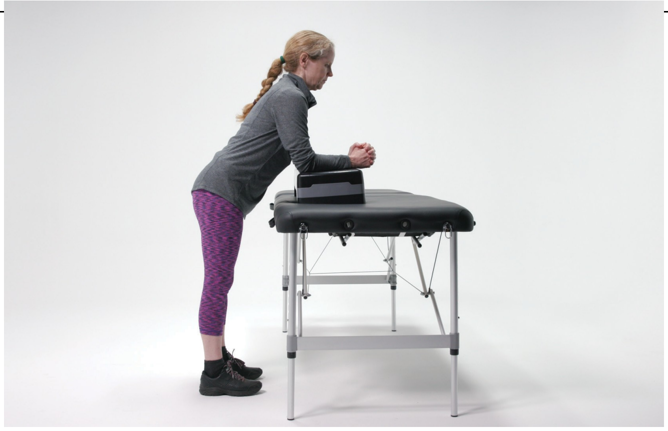

Exercise:

Bend your arthritis knee to around 90 degrees.

Push your foot backwards behind you so you are extending at your hip joint, while keeping your knee in the bent position.

Hold for 5 seconds then slowly return to the starting position.

Do 3 sets of 10 repetitions with a break of 30-60 seconds between sets.

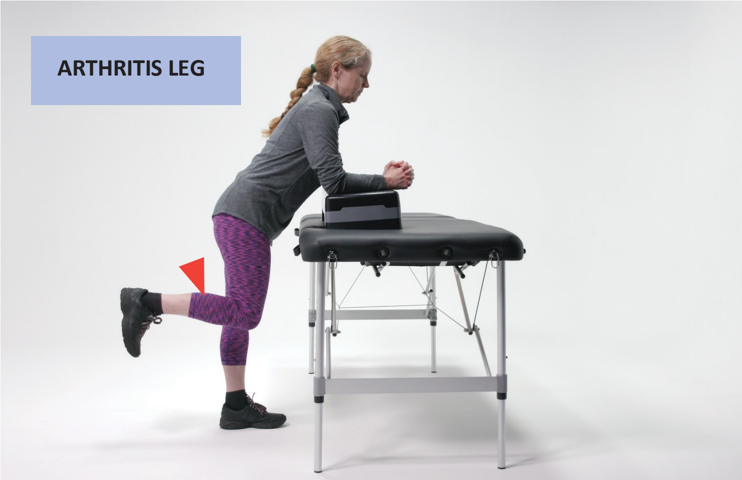

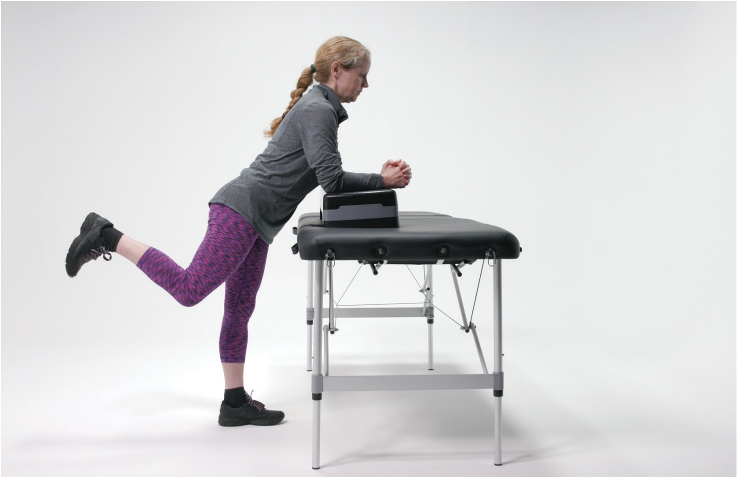

Exercise 7: Calf strengthening

Level 1: Double calf raises

Starting position:

Stand holding onto back of the chair.

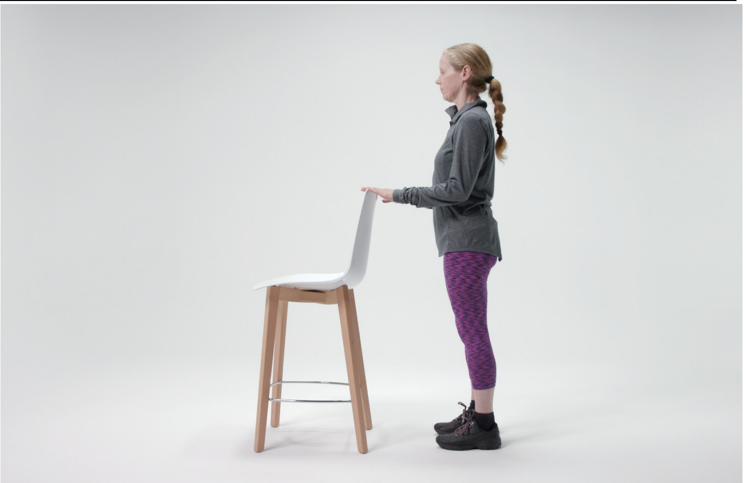

Exercise:

Slowly rise up onto your toes.

Hold for 5 seconds.

Slowly lower.

"Slowly up, hold, 2, 3, 4, 5, slowly down".

Do 3 sets of 10 repetitions with a break of 30-60 seconds between sets.

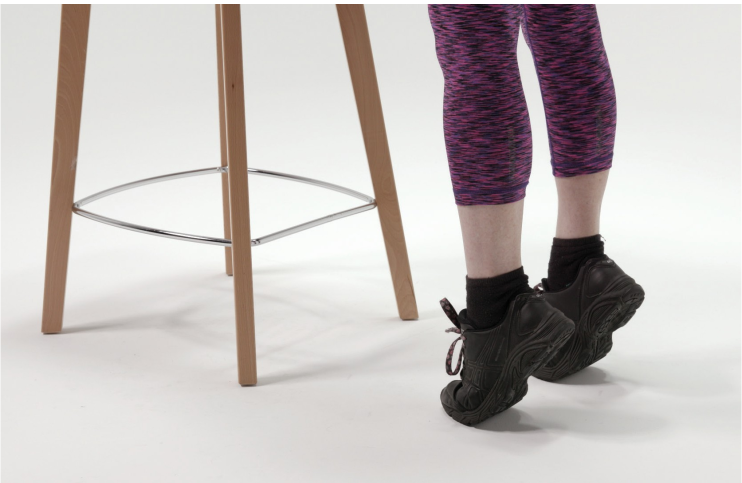

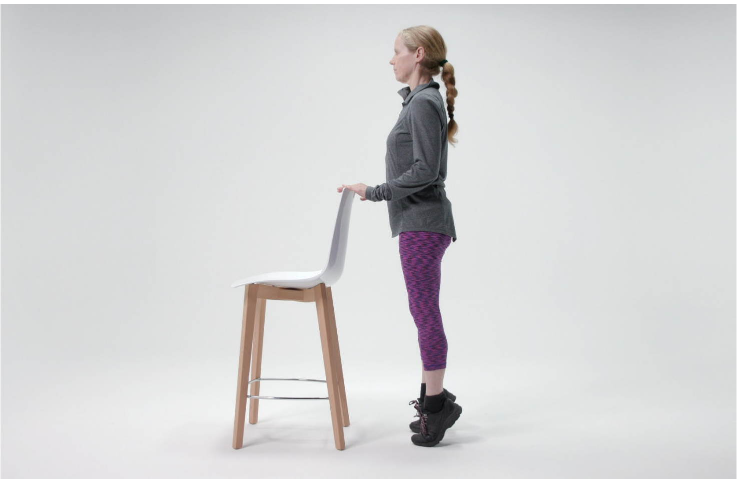

Exercise 7: Calf strengthening

Level 2: Single calf raises

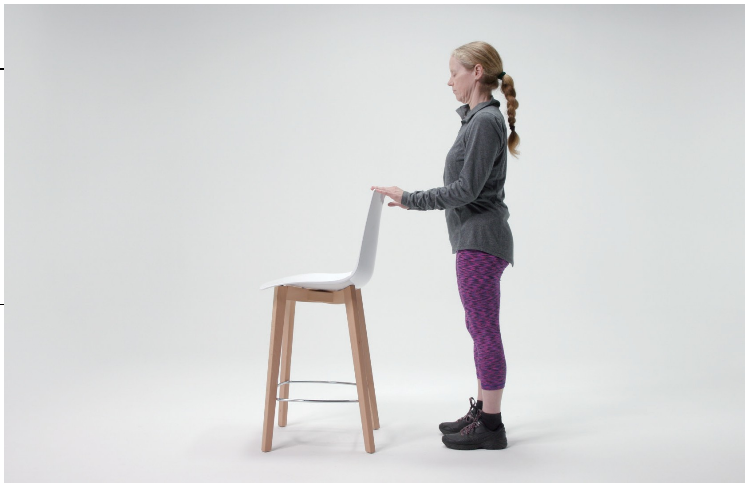

Starting position:

Stand holding onto back of the chair.
Stand on your arthritis leg.

Exercise:

Slowly rise up onto your toes. Hold for 5 seconds.

Slowly lower.

"Slowly up, hold, 2, 3, 4, 5, slowly down".

Do 3 sets of 10 repetitions with a break of 30-60 seconds between sets.

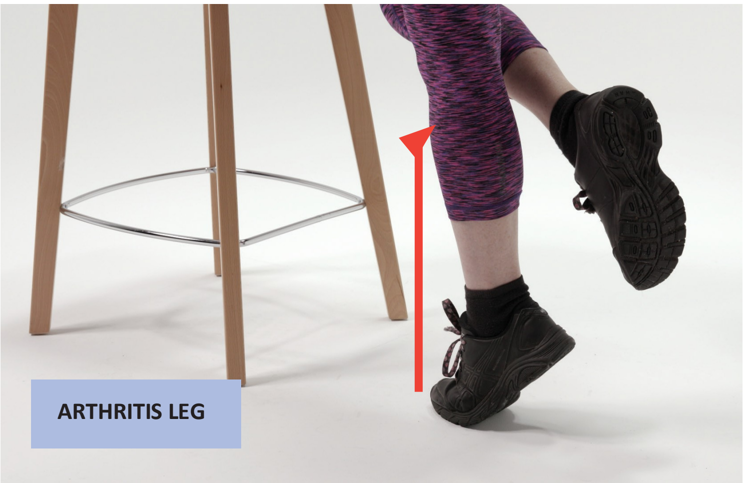

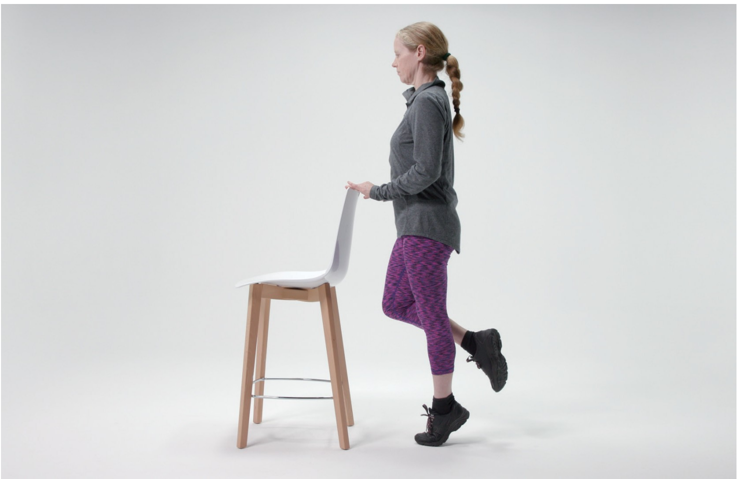

Exercise 7: Calf strengthening

Level 3: Double calf raises over edge of step

Starting position:

Stand on a step with your heels over the edge.
Hold onto the back of a chair for support.

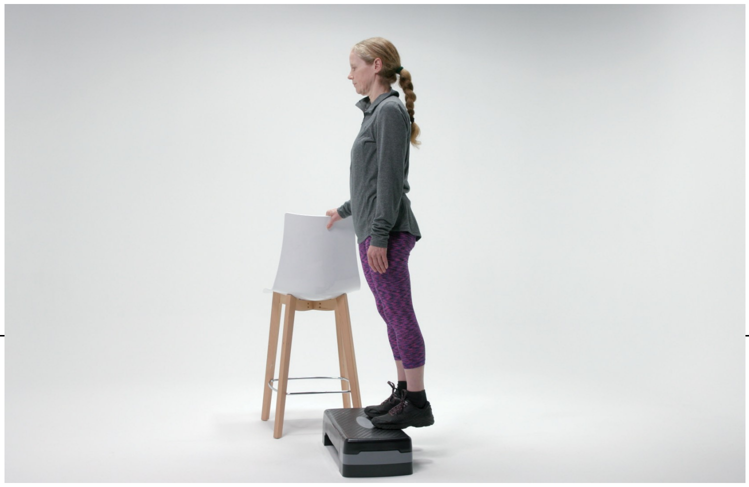

Exercise:

Slowly rise up onto your toes. Hold for 5 seconds.

Slowly lower down, so your heels are below the step.

"Slowly up, hold, 2, 3, 4, 5, slowly lower all the way down"..

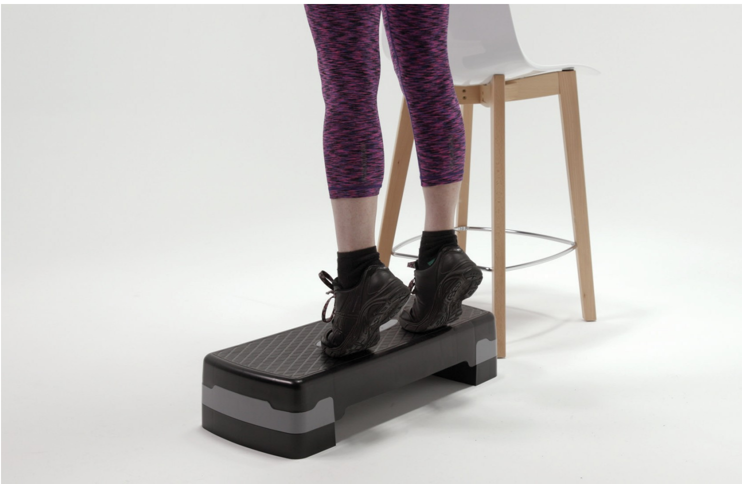

Do 3 sets of 10 repetitions with a break of 30-60 seconds between sets.

Exercise 7: Calf strengthening

Level 4: Single calf raises over edge of step

Starting position:

Stand on your arthritis leg on a step with your heel over the edge.
Hold onto the back of a chair for support.

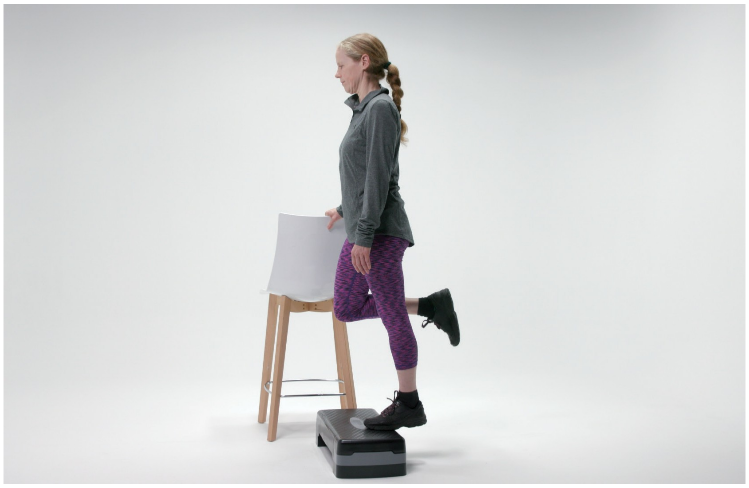

Exercise:

Slowly rise up onto your toes. Hold for 5 seconds.

Slowly lower down, so your heel is below the step.

"Slowly up, hold, 2, 3, 4, 5, slowly lower all the way down".

Do 3 sets of 10 repetitions with a break of 30-60 seconds between sets.

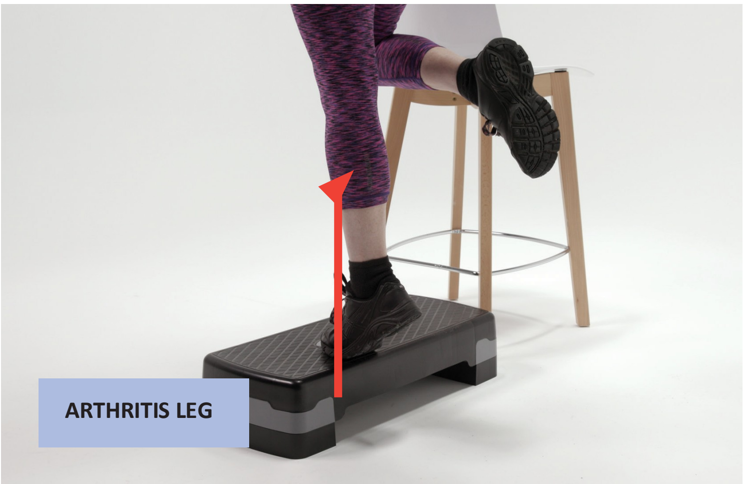

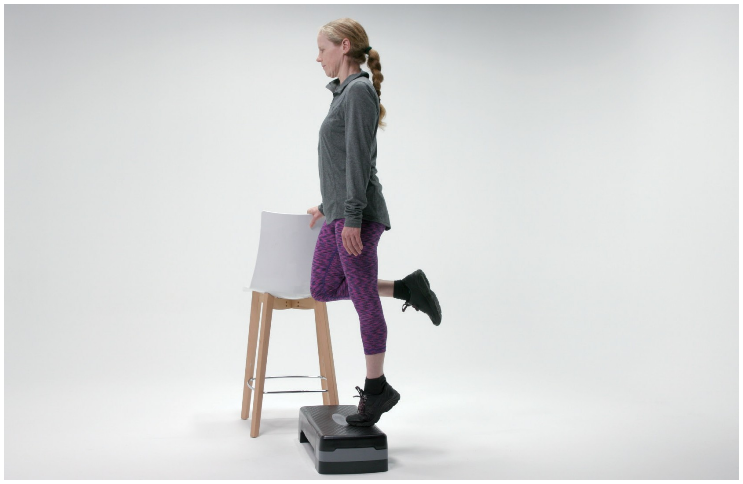

Exercise 8: Seated Knee Extension

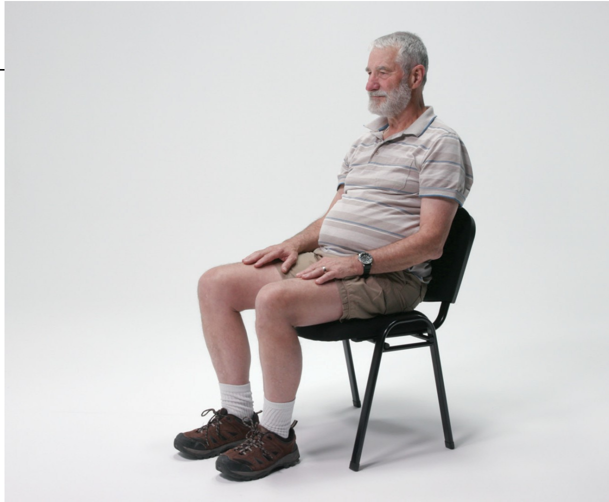

Starting position:

Sit in a firm chair (one that is higher if possible).

Exercise:

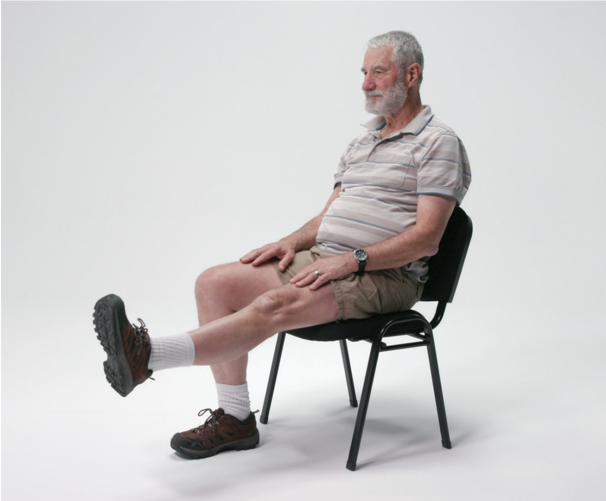

Slowly lift your foot up and straighten the knee until it is fully straight.

Keep the back of your thigh on the chair. Hold for 5 seconds and lower slowly.

“Slowly up, hold, 2, 3, 4, 5, slowly down”.

Progression:
Tie your elastic band into a loop.

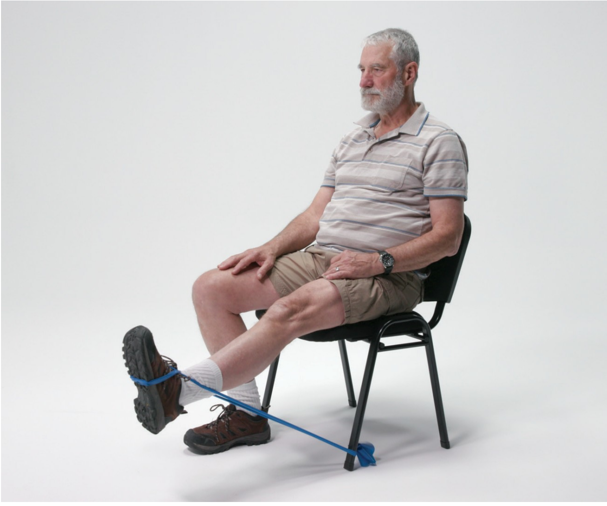

Place the looped elastic band around the back leg of a chair.

Sit on the chair and put your leg into the loop with the band around the front of your foot. Change colour of elastic band – red through to black.

Do 3 sets of 10 repetitions with a break of 30-60 seconds between sets.
